# A multi-item signal detection theory model for eyewitness identification

**DOI:** 10.1186/s41235-025-00652-3

**Published:** 2025-08-22

**Authors:** Yueran Yang, Janice L. Burke, Justice Healy

**Affiliations:** 1 Department of Psychology, University of Nevada, Reno, Reno, NV USA; 2 Interdisciplinary Social Psychology Ph.D. Program, University of Nevada, Reno, Reno, NV USA

**Keywords:** Eyewitness identification, Eyewitness memory, Eyewitness decision making, Signal detection theory

## Abstract

*How do witnesses make identification decisions when viewing a lineup?* Understanding the witness decision-making process is essential for researchers to develop methods that can reduce mistaken identifications and improve lineup practices. Yet, the inclusion of fillers has posed a pivotal challenge to this task because the traditional signal detection theory is only applicable to binary decisions and cannot easily incorporate lineup fillers. This paper proposes a multi-item signal detection theory (mSDT) model to help understand the witness decision-making process. The mSDT model clarifies the importance of considering the joint distributions of suspect and filler signals. The model also visualizes the joint distributions in a multivariate decision space, which allows for the incorporation of all eyewitness responses, including suspect identifications, filler identifications, and rejections. The paper begins with a set of simple assumptions to develop the mSDT model and then explores alternative assumptions that can potentially accommodate more sophisticated considerations. The paper further discusses the implications of the mSDT model. With a mathematical modeling and visualization approach, the mSDT model provides a novel theoretical framework for understanding eyewitness identification decisions and addressing debates around eyewitness SDT and ROC applications.

**Supplementary Information:**

The online version contains supplementary material available at 10.1186/s41235-025-00652-3.

## Introduction

Eyewitness memory provides critical information for police investigations (Albright, 2017; Wells et al., 2020). To collect this information, police often construct a lineup that consists of one suspect, who may be guilty or innocent, and several fillers, who are known to be innocent. When viewing a lineup, witnesses can identify the suspect, identify a filler, or reject the lineup. Yet, *how do witnesses use their memory to make identification decisions*? Understanding this decision-making process is vital for both the legal system and scholars to reduce mistaken identifications and improve lineup practices (National Research Council [NRC], 2014). 

Eyewitness researchers have applied a classical theoretical framework in recognition memory—signal detection theory (SDT)—to understand eyewitness identification decisions (e.g., Meissner et al., 2005; Palmer & Brewer, 2012; Wixted & Mickes, 2014). This application, however, is not without complications. One major controversy arises from the multiple recognition items involved in eyewitness lineup tasks: a suspect, who may be guilty or innocent, and several fillers (usually five). The inclusion of fillers complicates the application of SDT to eyewitness identification decisions because eyewitness responses do not conform to the binary decisions that SDT typically deals with (Lampinen, 2016; Wells et al., 2015).

Several modified signal detection models have been developed to incorporate lineup fillers (e.g., Clark, 2003; Duncan, 2006; Lee & Penrod, 2019; Wixted et al., 2018). These models have built an important theoretical foundation for understanding eyewitness identification decisions. Yet, previous models generally present eyewitness lineup tasks in a univariate decision space, which is only suitable for the classic SDT tasks that present a single recognition item for each decision. As noted above, lineup tasks involve more than one recognition item (i.e., one suspect and several fillers). Modeling signals of these items in a univariate space may eliminate important information, such as the relations among lineup members and the complexity of witness decision rules, thereby hindering a proper understanding of eyewitness identification decisions.

This paper proposes a multi-item signal detection theory (mSDT) model to better understand eyewitness identification decisions. A key feature of the mSDT model is that it considers these decisions within the framework of *joint* distributions of suspect and filler signals. The mSDT model mathematically derives these joint distributions and visualizes them in a multivariate decision space. This approach enables the model to account for the relations among lineup members and map complex witness decision rules onto the decision space, thereby incorporating all three eyewitness responses (suspect identifications, filler identifications, and rejections). Through a mathematical modeling and visualization approach, the mSDT model offers an important theoretical framework for understanding eyewitness lineup tasks and analyzing how various factors affect eyewitness decisions.

The paper is organized as follows: The first section briefly reviews signal detection theory and its application to eyewitness decisions. The next two sections introduce the mSDT model and discuss key differences between the mSDT and the classic univariate SDT models. The subsequent section explores potential model variants. Finally, the last section addresses the theoretical and practical implications of the mSDT model.

## Signal detection theory and application to eyewitness decisions

**Fig. 1:**
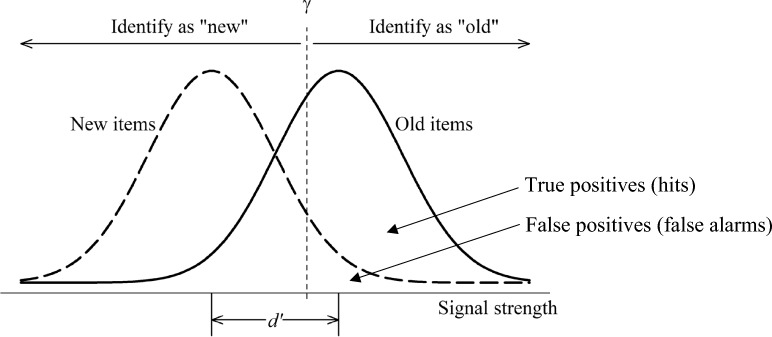
A Signal Detection Model for Item Recognition. *Note*: The dashed curve depicts the distribution of new items, and the solid curve depicts the distribution of old items. The vertical dashed line depicts the decision criterion $\gamma$

Signal detection theory (SDT) provides an indispensable theoretical framework for understanding and analyzing human recognition memory (Yonelinas & Parks, 2007). In a classic old-new recognition task, people view either an old or a new item and judge if they have seen the item before. This is similar to a showup, in which witnesses view either a guilty or an innocent suspect, without any fillers. SDT models people’s memory signals for old and new items as two distributions, typically assumed to be normal. The old-item distribution has a higher mean than the new-item distribution, meaning that people generally have stronger memory for old items than new ones. When deciding whether an item is old or new, people compare the item’s signal strength with a decision criterion. If the signal strength exceeds the criterion, the item is identified as “old”; otherwise, it is identified as “new” (see Fig. 1).

According to SDT, people’s recognition decisions result from the combination of two parameters: *discriminability* (denoted as *d’*), which quantifies the mean distance between the old and new signal distributions, and *decision criterion* (denoted as *γ*), which quantifies the criterion adopted for making a recognition decision (Macmillan & Creelman, 2005). Many researchers have used the letter *c* to denote the decision criterion. This paper uses *γ* to denote the decision criterion to avoid a confusion between the decision criterion and a cost function, which is commonly used in evaluating lineup performance and denoted as *c* (e.g., Yang et al., 2019). Because discriminability reflects the properties of the old and new distributions and is not influenced by changes in decision criterion, it has been used as a primary measure for quantifying performance (Swets, 1988; Swets et al., 2000). 

As one of the most successful applications of mathematical modeling in psychology, SDT also serves as a valuable tool for analyzing recognition data (Luce, 1995; Swets, 1996). As shown in Fig. 1, the true-positive rate (or hit rate)—the probability that people correctly recognize *old* items as “old”—corresponds to the area under the old-item distribution beyond the decision criterion. The false-positive rate (or false alarm rate)—the probability that people mistakenly recognize *new* items as “old”—corresponds to the area under the new-item distribution beyond the decision criterion. According to SDT, these two rates change when people adjust their decision criterion. With a liberal criterion, both true- and false-positive rates will be high; with a conservative criterion, both rates will be low. This trade-off between true- and false-positive rates is often visualized using receiver operating characteristic (ROC) curves, which plot true-positive rates against false-positive rates at all levels of decision criteria (Green & Swets, 1966; Macmillan & Creelman, 2005).

### SDT application to eyewitness decisions

Because of its success in modeling recognition tasks, researchers have applied SDT to eyewitness lineup tasks, which also involve recognition memory (e.g., Gronlund et al., 2012; Wixted & Mickes, 2014). When viewing a lineup, witnesses rely on their memory to decide if any of the lineup members match their recollection of the culprit. In laboratory-based experiments, witnesses are randomly assigned to view either culprit-present or culprit-absent lineups. Accordingly, their decisions are often recorded in a 3 × 2 contingency table, which captures the six possible identification outcomes (see Table 1).

**Table 1:** Eyewitness Identification Outcomes in Culprit-Present and Culprit-Absent Lineups

		Witness response		
		Suspect identification	Filler identification	Rejection
**Lineup status**	**Culprit present (suspect being guilty)**	$\Pr (IDS|G)$	$\Pr (IDF|G)$	$\Pr (REJ|G)$
	**Culprit absent (suspect being innocent)**	$\Pr (IDS|I)$	$\Pr (IDF|I)$	$\Pr (REJ|I)$

In Table 1, the letters *G* and *I* denote suspects’ guilt status as either guilty or innocent (i.e., lineup status being culprit present or absent). The abbreviations *IDS*, *IDF*, and *REJ* denote witnesses’ three possible responses—identify the suspect, identify a filler, and reject the lineup. Because identification outcomes are contingent upon suspects’ guilt status, they are denoted as conditional events, *Response|Truth* (read as *response given truth*). For example, *IDS|G* denotes identifying the suspect given that the suspect is guilty (i.e., in a culprit-present lineup), and *REJ|I* denotes rejecting the lineup given that the suspect is innocent (i.e., in a culprit-absent lineup). The function $Pr\left(*|*\right)$ denotes the probability of an identification outcome.

It is important to note that this 3 × 2 data structure from eyewitness laboratory experiments does not conform to the 2 × 2 binary recognition tasks typically handled by SDT. This incompatibility is caused by the multiple recognition items involved in a lineup—witnesses view not only one suspect *but also several fillers*. To incorporate lineup fillers, researchers have developed several modified SDT models, such as the WITNESS model (Clark, 2003), the compound signal detection model (Duncan, 2006), the Ensemble model (Wixted et al., 2018), and the multi-*d’* signal detection theory model (Lee & Penrod, 2019). Below we briefly review each model.

#### The WITNESS model

The WITNESS model was developed as a computer simulation framework that can implement any eyewitness identification model (Clark, 2003). The WITNESS model first simulates suspect and filler signals and then predicts witness responses according to two types of decision rules: absolute and relative judgements (Wells, 1984). The model incorporates these two types of judgments in the form of a weighted sum of signals (or match values) that represent how well lineup members match the recollection of the perpetrator (Clark, 2003). Absolute judgments are based on the signal of the best-matching lineup member (i.e., the BEST decision rule), whereas relative judgments are based on the difference in signals between the best and next-best lineup members (i.e., the DIFF decision rule).

The WITNESS model predicts that an identification of the best-matching lineup member will be made if the weighted sum of the best match (absolute judgment) and the difference between the best and next-best match (relative judgment) is greater than a decision criterion. A rejection will be made if all matches are less than a rejection criterion. In addition, the model incorporates the “do not know” response by distinguishing the decision criterion and the rejection criterion. The WITNESS model provides an important framework for modeling and simulating witness responses under different considerations.

#### The compound signal detection model

The compound signal detection (SDT-CD) model conceptualizes a lineup task as a compound decision-making process that involves two steps: a detection task and an identification task (Duncan, 2006). In the detection task, witnesses determine whether the culprit is present or not. Once the culprit is detected to be present, witnesses then engage in the identification task and pick out the target among all lineup members. For the detection task, the SDT-CD model discusses two types of decision rules: the independent observation rule and the integration rule. The independent observation rule states that witnesses will make a positive detection if at least one lineup member’s memory signal exceeds the response criterion; the integration rule states that witnesses will make a positive detection if the sum of all lineup members’ memory signals exceeds the response criterion. For the identification task, the decision rule is to choose the lineup member with the strongest signal. With the multiple steps involved in a compound decision task, the SDT-CD model seeks to better understand eyewitness decisions.

#### The Ensemble model

The Ensemble model proposes that witnesses make decisions based on the difference between an individual lineup member’s signal and the average signal of all lineup members (Wixted et al., 2018). In other words, witnesses’ decision variable is a lineup member’s difference score from the average signal strength. Similar to the BEST rule in the WITNESS model, the Ensemble model uses the MAX decision rule, which states that the lineup member with the strongest signal will be identified if its difference score exceeds a decision criterion. In addition, the Ensemble model emphasizes the importance of considering correlated signals because faces in a lineup inevitably share features (Wixted & Mickes, 2014). The model predicts that discriminability enhances when the correlation among lineup member signals increases.

#### The multi-d’ signal detection theory model

The multi-*d’* signal detection theory (multi-*d’* SDT) proposes different measures of discriminability due to the involvement of fillers (Lee & Penrod, 2019). Particularly, the model considers three distributions—a guilty suspect distribution, an innocent suspect distribution, and filler distributions. Based on these distributions, the model calculates multiple discriminability measures, including the discriminability of guilty from innocent suspects, guilty suspects from fillers, innocent suspects from fillers, and fillers in culprit-present lineups from fillers in culprit-absent lineups. The model shows that the discriminability of guilty from innocent suspects can be decomposed into other discriminability measures involving fillers, highlighting the importance of fillers in a lineup task.

### Controversies around eyewitness SDT applications

The above eyewitness models have provided important insight and built a strong theoretical foundation for understanding eyewitness identification decisions. Particularly, the idea that using discriminability as a primary measure for quantifying lineup performance has been highly applauded and widely adopted by scholars and practitioners (NRC, 2014).

Because of the involvement of lineup fillers, however, the relationship between eyewitness SDT tasks and ROC curves has remained obscure, and their applications to lineup tasks have sparked considerable controversy. Two major issues stand out. As aforementioned, the first issue concerns the 3 × 2 data structure of witness responses in laboratory-based experiments. As noted, witnesses view multiple items (one suspect and several fillers) in a lineup task, yielding responses that fall into a 3 × 2 data table (see Table 1). It remains ambiguous how this 3 × 2 structure could conceptually align with the traditional 2 × 2 data structure generated from standard binary recognition tasks.

The second issue concerns applying ROC analysis to lineup data. Because eyewitness responses do not align with the 2 × 2 data structure of binary recognition tasks, researchers typically create “partial” ROC curves by exclusively plotting suspect identification rates at different confidence levels (e.g., Wixted & Mickes, 2012). However, critics point out that this practice focuses solely on suspect identifications, neglecting filler identifications and rejections. As they overlook the complete picture of eyewitness responses, “partial” suspect-only ROC curves may not accurately reflect witness discriminability (Lampinen, 2016; Wells et al., 2015). Even for the recently developed full ROC curves that can incorporate all eyewitness responses, it remains debatable whether these curves reflect witness discriminability (i.e., how well witnesses can distinguish between guilty and innocent suspects) or investigator discriminability (i.e., how effectively investigators can use eyewitness evidence to judge suspects’ guilt or innocence) (Smith et al., 2020; Yang & Smith, 2023).

We believe that these controversies stem in part from the imprecise presentation of lineup tasks. Previous eyewitness SDT models generally project *separate* filler distributions onto the same univariate space used in a binary recognition task, which includes a guilty suspect distribution (i.e., the old-item distribution) and an innocent suspect distribution (i.e., the new-item distribution). Although the classic univariate SDT (uSDT) model is highly useful for understanding tasks that involve single recognition items, it becomes less precise when modeling lineup tasks that involve *multiple* recognition items, potentially causing confusion. For example, by treating the joint distribution of lineup member signals as separate univariate distributions, uSDT fails to capture the relations among these signals. Similarly, by using a univariate space, uSDT distorts nonlinear decision rules into linear ones. A more detailed discussion on the data-model discrepancies when applying uSDT to eyewitness decisions is included in the supplementary materials.

A rigorously defined mathematical model that could properly incorporate all lineup members would greatly enhance the understanding of eyewitness identification decisions, just like the classic SDT benefits the understanding of binary recognition decisions. The current paper intends to fill this gap.

## A multi-item signal detection theory model

This paper proposes a multi-item signal detection theory (mSDT) model to understand eyewitness identification decisions. The mSDT model has two unique features. First, it constructs a rigorously defined mathematical model that examines the joint distributions of both suspect and filler signals. The joint distributions more accurately capture witness decision variables in a lineup task—one for lineup member signals in culprit-present lineups and the other for those in culprit-absent lineups. The mSDT model first develops the joint distributions from a set of simple assumptions. The model further explores potential assumptions that can accommodate more sophisticated considerations such as unequal variances, correlated signals, and alternative decision rules. Identifying and modeling the proper joint distributions for a lineup task has important implications for how to estimate model parameters and understand eyewitness decisions, particularly with the presence of fillers.

Beyond *mathematically* modeling the joint distributions, another contribution of the mSDT model is the *visual* representation of the mathematical model. The mSDT model *visually* presents the witness decision-making process in a multivariate decision space. The visual format of the model is profoundly important because visualizations allow for a deeper comprehension of complex concepts in a more structured way (Keller & Tergan, 2005; Palais, 1999). Particularly, the visualization shows not only how the joint distributions incorporate both suspect and filler signals, but also how witness decision rules partition the joint distributions into segments that correspond to different eyewitness identification outcomes. In this paper, we primarily present the visual format of the model due to its ease of understanding and include most of the mathematical derivations in the appendices.

Before introducing the mSDT model, we define key concepts and introduce assumptions involved in the model. We first focus on a set of simple assumptions for ease of understanding. In later sections, we discuss alternative assumptions that can accommodate more sophisticated considerations.

### Model notations

1. ${ss}_{i}$ denotes the suspect’s signal for the *i*^*th*^ witness (*ss* stands for *suspect signal*)*.* If the suspect is guilty, the signal is denoted as ${ss}_{i}|G$ (suspect signal given guilt). If the suspect is innocent, it is denoted as ${ss}_{i}|I$ (suspect signal given innocence). The means of innocent and guilty suspect signals are denoted as ${\mu }_{I}$ and ${\mu }_{G}$, and the variances as ${\sigma }_{I}^{2}$ and ${\sigma }_{G}^{2}$, respectively.
2. ${fs}_{ij}$ denotes the *j*^*th*^ filler’s signal for the *i*^*th*^ witness (*fs* stands for *filler signal*)*.* The mean of filler signals is denoted as ${\mu }_{F}$ and the variance as ${\sigma }_{F}^{2}$. Among all fillers in a lineup, ${fs}_{imax}$ denotes the filler with the strongest signal, that is, ${fs}_{imax}=\text{max}\left({fs}_{ij}\right)$.
3. $\gamma$ denotes the decision criterion witnesses use to make an identification decision. As will be described in model assumptions, witnesses will identify a lineup member if it has the strongest signal and exceeds $\gamma$, and will reject a lineup if the strongest signal does not exceed $\gamma$.

### Model assumptions

1. Witnesses rely on lineup member signals for making identification decisions. In other words, the signals of all lineup members constitute the decision variable when witnesses view a lineup that simultaneously presents all of them.
2. The signals of guilty suspects, innocent suspects, and fillers are random samples drawn from independent normal distributions with the same variance (for simplicity, the variances are assumed to be one). In other words, the model assumes random sampling, independence, normal distribution, and equal variance for all lineup member signals. Thus, these lineup member signals are assumed to follow probability distributions, allowing for a quantitative analysis of witness decision-making processes.
3. The innocent suspect distribution has the same mean as the filler distribution in a fair lineup. These means are conventionally set to zero, ${\mu }_{I}={\mu }_{F}=0$. Accordingly, an innocent suspect would have an equal chance of being identified as any of the fillers; that is, none of the lineup members would stand out, resulting in a fair lineup. The guilty suspect distribution has a greater mean compared to other distributions, ${\mu }_{G}>{\mu }_{I}={\mu }_{F}=0$. In other words, witnesses generally have stronger memory for a guilty suspect than an innocent suspect or fillers. Similar to the classic uSDT, witness discriminability is defined as the standardized mean difference between guilty and innocent distributions, denoted as ${d}_{GI}^{\prime}.$ In this context, witness discriminability reflects how effectively witnesses can distinguish between guilty and innocent suspects. Given that the distribution variances are set to one and the mean of the innocent suspect distribution is set to zero, we will have ${\mu }_{G}={d}_{GI}^{\prime}$.

Using mathematical symbols, assumptions 1–3 can be expressed as:

$${ss}_{i}|G \overset{\mathrm{i.i.d.}}{\sim} \mathcal{N} ({d}^{\prime}_{GI}, 1)$$

$${ss}_{i}|I \overset{\mathrm{i.i.d.}}{\sim} \mathcal N (0, 1)$$

$${fs}_{ij} \overset{\mathrm{i.i.d.}}{\sim} \mathcal N(0, 1)$$

In the above expressions, *i.i.d.* stands for “independent and identically distributed.”

(4) Witnesses use the MAX decision rule to make identification decisions (Clark, 2003; Wixted et al., 2018). Specifically, witnesses judge which lineup member yields the strongest signal and then compare the strength of that signal to their decision criterion. When the strongest signal exceeds the criterion, witnesses affirmatively identify that lineup member. When the strongest signal does not exceed the criterion, witnesses reject the lineup.

**Fig. 2:**
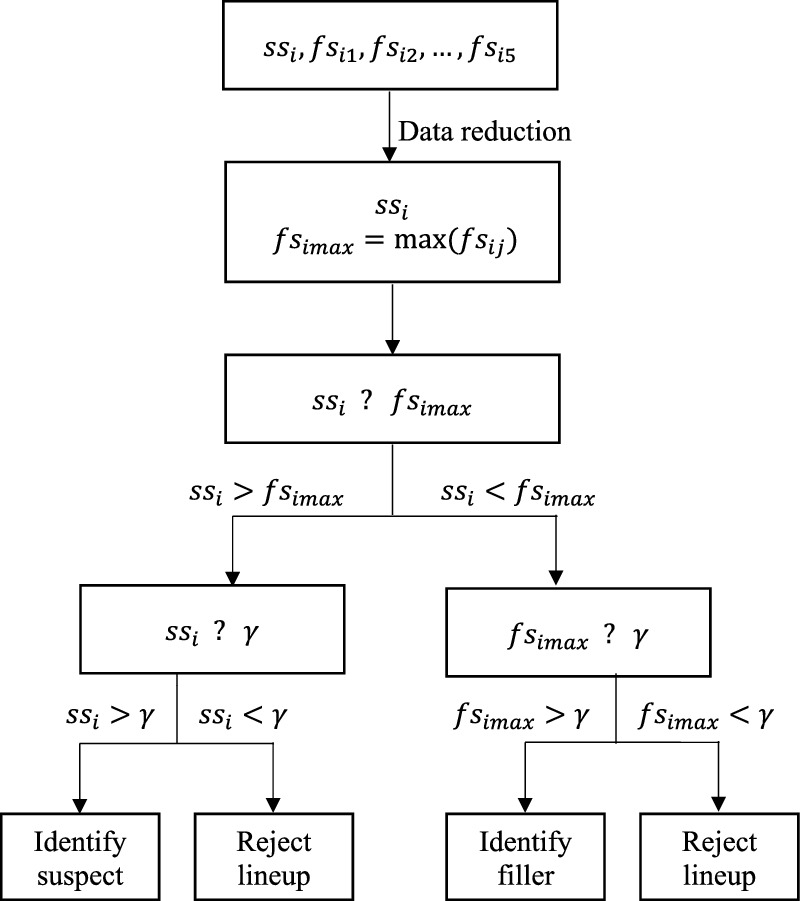
Decision Tree for Eyewitness MAX Decision Rule. *Note:* *ss*_*i*_ denotes the signal of a suspect in the lineup for the *i*^*th*^ witness. *ss*_*i*_ could come from either a guilty suspect distribution (i.e., a culprit-present lineup) or an innocent suspect distribution (i.e., a culprit-absent lineup). *fs*_*ij*_ denotes the signal of the *j*^*th*^ filler in the lineup, and *fs*_*imax*_ denotes the filler with the strongest signal (“max filler”). *γ* denotes witnesses’ decision criterion

The decision tree in Fig. 2 depicts the process of how witnesses make different responses according to the MAX decision rule. The decision tree does not specify whether the suspect signal comes from a guilty or an innocent suspect because the same process applies to both culprit-present and culprit-absent lineups. Nevertheless, the decision tree does differentiate between suspect and filler signals. Although witnesses do not distinguish between suspects and fillers during their decision process because they do not know lineup members’ identities, police investigators do know lineup members’ identities and thus would treat suspect identifications and filler identifications differently (Smith et al., 2020; Yang & Smith, 2023). To incorporate both views, the decision tree differentiates between suspect identifications and filler identifications in the witness decision-making process.

(5) In eyewitness research, witness confidence ratings are often used as a proxy for decision criteria (Wixted & Mickes, 2014). This common practice entails several implicit assumptions. First, the proxy assumes all witnesses use the same decision criteria (i.e., no criterion variability; Smith et al., 2017). For example, when witnesses report “high” confidence in their identifications, they all use the same criterion $\gamma =2$. Second, this proxy assumes the same confidence rating reflects the same decision criterion across culprit-present and culprit-absent lineups (i.e., no criterion shift). For example, a “high” confidence rating corresponds to the same criterion $\gamma =2$ across both culprit-present and culprit-absent lineups. Last, but most importantly, this proxy assumes that witnesses use the same type of decision rules for making identification decisions and rating confidence, which we refer to as the “uniform decision rule” assumption. We elaborate on this assumption later.

### Max filler signal distribution

**Fig. 3:**
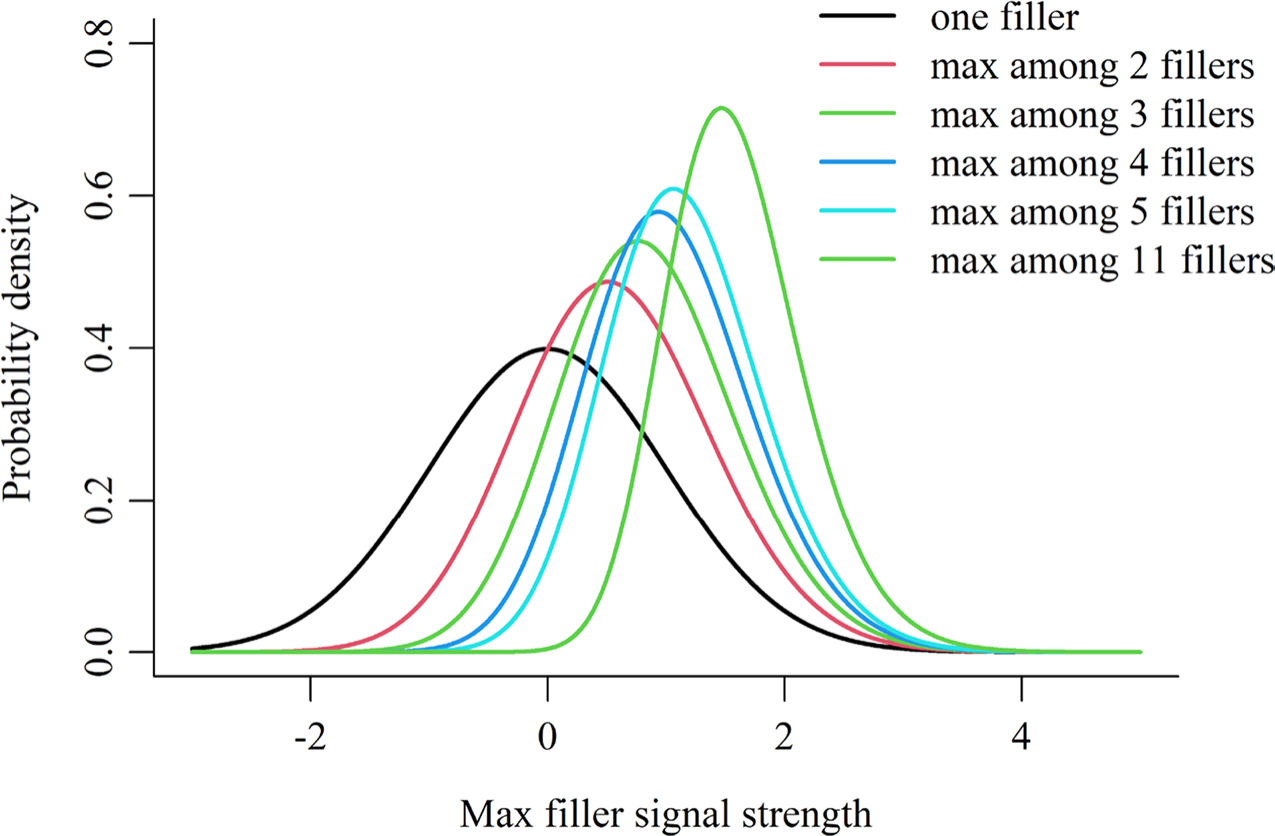
Probability Distributions of Max Filler Signals in Lineups with Different Sizes

The MAX decision rule implies that witnesses’ decision process involves the filler with the strongest signal (“max filler”). Therefore, multiple filler signals in a lineup can be reduced to one—the max filler signal. Understanding the distribution of max filler signals is important for understanding the role of fillers. Figure 3 visualizes the probability density distributions of the max filler signals in different-sized lineups (lineup size 2–6 and 12; or equivalently, filler size 1–5 and 11). Note that the max filler signals do not follow a normal distribution, though individual fillers do follow a normal distribution as assumed in the model (see Appendix A for the derivations of the max filler probability density function).

Several properties of the max filler distributions are worth noting. First, the distributions are not symmetric (except for the one filler distribution, which follows a normal distribution). The distributions are positively skewed, meaning that they have slightly longer tails in the positive (right) direction. This asymmetry is more apparent in the contour plots shown in the next section. Second, as the number of fillers increases, the mean of the distribution increases and the variance decreases. With more fillers, the chance that one of the fillers will produce a very strong signal increases, thus leading to a greater max value; at the same time, large fluctuations become less likely, stabilizing the max value and thus reducing the variance.

The closed-form expressions of the mean and variance of the max filler distribution are not easy to derive (David & Nagaraja, 2003). Therefore, we display their numeric approximations in Appendix A (see Table A1). The R code for plotting the density curves and calculating the descriptive statistics of the max filler distributions is available at osf.io/n2zbc/**.**

### Joint distributions of suspect and max filler signals

Because a lineup contains not only a suspect but also fillers, one must consider a joint distribution that can incorporate all lineup member signals. As discussed above, multiple filler signals can be reduced to one—the max filler signal. Therefore, such a joint distribution needs at least to incorporate both suspect signals and max filler signals. We first focus on culprit-present lineups and discuss the joint distribution of guilty suspect and max filler signals. We then turn to culprit-absent lineups and discuss the joint distribution of innocent suspect and max filler signals.

#### Joint distribution for culprit-present lineups

**Fig. 4:**
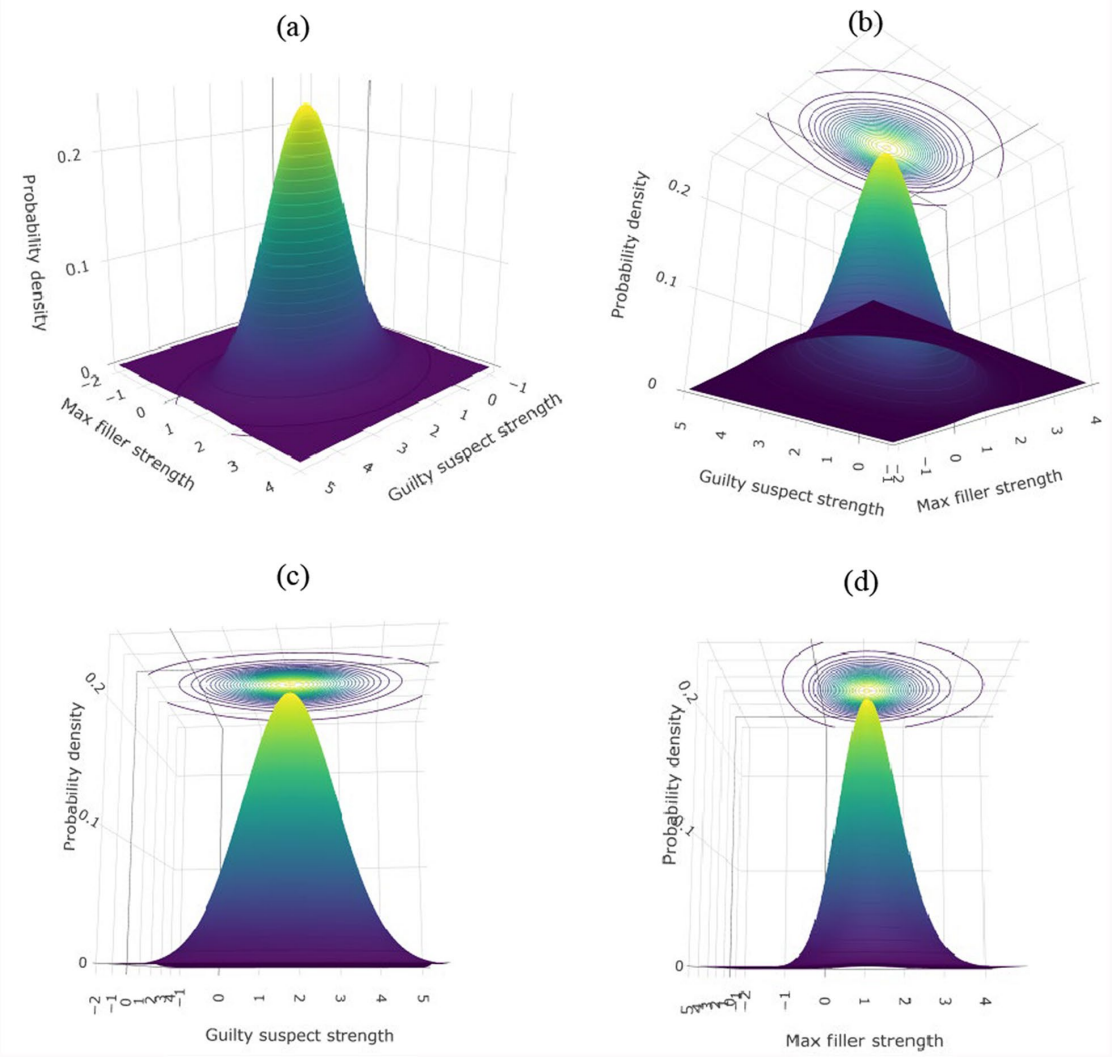
Joint Distribution of Guilty Suspect and Max Filler Signals

In culprit-present lineups, witnesses’ signal detection task can be captured by a joint distribution of *guilty* suspect and max filler signals. Under the independence assumption, this joint distribution can be derived from multiplying the marginal distributions of these signals. Figure 4 depicts such a joint distribution. The *x*-axis represents guilty suspect signals, the *y*-axis represents max filler signals, and the *z*-axis represents probability density of the joint distribution. In this example, the lineup consists of six members (one guilty suspect and five fillers), with the mean of guilty suspect signals set to two (i.e., ${d}_{GI}^{\prime}=2$).

All four panels in Fig. 4 show different rotations of the same joint distribution. Panel (c) rotates to show the marginal distribution of guilty suspect signals, which is a normal distribution ($ss|G \sim \mathcal N(2, 1)$), and panel (d) rotates to show the marginal distribution of max filler signals, which is the same as the max filler distribution in Fig. 3. Interested readers can go to osf.io/n2zbc/for a rotatable animation of the joint distribution.

**Fig. 5:**
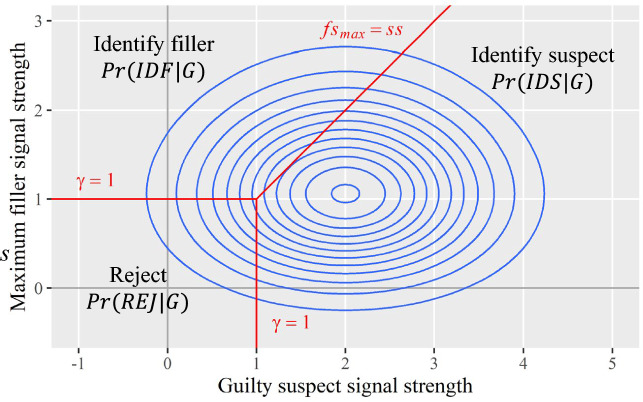
Contour Plot for the Joint Distribution of Guilty Suspect and Max Filler Signals. *Note:* The blue contours depict the joint distribution of guilty suspect and max filler signals for culprit-present lineups. The red lines depict the MAX decision rule

To facilitate readers’ understanding, we map the three-dimensional joint distribution onto a contour plot (see Figs. 4b–d and 5). In the contour plot, the *x-* and *y-*axes are the same as those used in the three-dimensional coordinates, with the *x*-axis representing guilty suspect signals and the *y*-axis representing max filler signals. The contours capture the information on the *z-*axis in the three-dimensional coordinates by connecting the (*x*, *y*) points that have the same probability density (i.e., the same height). Therefore, the contour plot retains the same information as the three-dimensional space.

The joint distribution in the contour plot also provides useful information on the marginal distributions. For example, the *x*-axis direction reflects the marginal distribution of guilty suspect signals. Readers can observe that the mean of the marginal distribution is two and the distribution is symmetric around its mean. Similarly, the *y*-axis direction reflects the marginal distribution of max filler signals. As discussed above, this marginal distribution is not symmetric. The mean of the max filler distribution is bigger than the mean of a single filler distribution, and the variance is smaller. Specifically, the mean of the max filler distribution is 1.16 and the variance is 0.45 (also see Table A1 in Appendix A).

Most importantly, the MAX decision rule can be mapped onto the multivariate decision space. In the original three-dimensional space, the MAX decision rule exists as three two-dimensional vertical planes, all perpendicular to the *x–y* plane. In the contour plot, these planes project onto the *x–y* plane as lines, shown as the three red lines in Fig. 5. Specifically, the vertical and horizontal red lines represent witnesses’ decision criterion, which is set arbitrarily as $\gamma =1$ in this example. The oblique red line represents the situation when suspect signal and max filler signal are the same (i.e., line of equality, ${fs}_{max}=ss$).

As shown in Fig. 5, the MAX decision rule partitions the multivariate decision space into three regions. Witnesses will identify the suspect if the suspect signal exceeds both the max filler signal and the decision criterion (the region to the right of the equality line ${fs}_{max}=ss$ and the vertical criterion line $\gamma =1$). Witnesses will identify the max filler if the max filler signal exceeds both the suspect signal and the decision criterion (the upper left region to the left of the equality line ${fs}_{max}=ss$ and beyond the horizontal criterion line $\gamma =1$). Witnesses will reject the lineup if neither the suspect nor max filler signals exceed the decision criterion (the lower left region). These three regions thus partition the joint distribution into three segments. The volume of each segment corresponds to the probability of each of the three eyewitness outcomes in *culprit-present* lineups (first row in Table 1).

#### Joint distribution for culprit-absent lineups

**Fig. 6:**
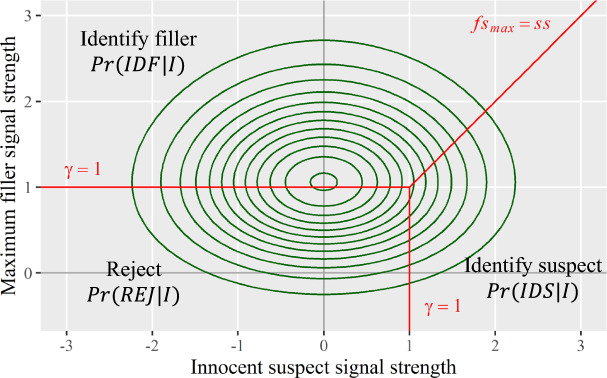
Contour Plot for the Joint Distribution of Innocent Suspect and Max Filler Signals. *Note:* The green contours depict the joint distribution of innocent suspect and max filler signals for culprit-absent lineups. The red lines depict the MAX decision rule

In culprit-absent lineups, witnesses’ signal detection task can be captured by a joint distribution of *innocent* suspect and max filler memory signals. Figure 6 displays this joint distribution in a contour plot. Note that for this joint distribution, the mean of innocent suspect signals is zero. Same as the joint distribution for culprit-present lineups, this joint distribution is also partitioned into the three segments by the MAX decision rule. The volume of each segment corresponds to the probability of each of the three eyewitness outcomes in *culprit-absent* lineups (second row in Table 1).

Interested readers can check Appendix B for how to derive the probabilities of different eyewitness outcomes in culprit-absent and culprit-present lineups. These derivations are important for the inverse operation—estimating discriminability and decision criterion from probabilities of eyewitness outcomes (i.e., eyewitness response rates), which will be discussed below.

#### Overlay joint distributions for culprit-present and culprit-absent lineups

**Fig. 7:**
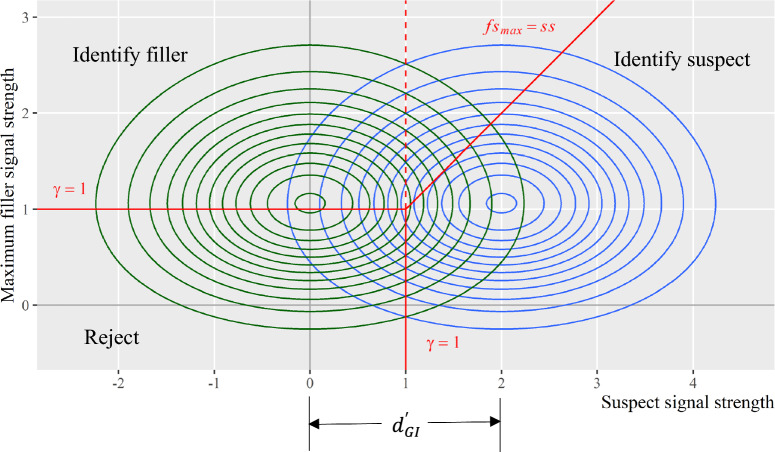
Joint Distributions for Culprit-Present Lineups and Culprit-Absent Lineups. *Note:* The blue contours depict the joint distribution of guilty suspect and max filler signals for culprit-present lineups. The green contours depict the joint distribution of innocent suspect and max filler signals for culprit-absent lineups. The red lines depict the MAX decision rule. The discriminability ${d}_{GI}^{\prime}$ represents the distance between the marginal means of the two joint distributions in the *x*-axis direction

Figure 7 depicts the joint distributions for culprit-present and culprit-absent lineups in the same plot. The three red lines show the MAX decision rule and assume witnesses would hold the same response criterion $\gamma =1$ in both culprit-present and culprit-absent lineups. Same as Figs. 5 and 6, the MAX decision rule partitions each distribution into three segments, corresponding to the three eyewitness outcomes under each lineup status. Therefore, the mSDT model effectively explains the 3 × 2 data structure produced by eyewitness responses.

Figure 7 also visualizes the discriminability parameter, ${d}_{GI}^{\prime}$, which is the mean distance between the marginal distributions of guilty and innocent suspect signals. In other words, it is the mean distance between the two joint distributions in the *x-*axis direction. Under the MAX decision rule, one can use the following equations to estimate witnesses’ decision criterion *γ* and discriminability ${d}_{GI}^{\prime}$ from eyewitness response rates.

## Equation 1.1. Decision criterion*** γ***

$$\gamma ={\Phi }^{-1}\left[\sqrt[m]{\text{Pr}\left(REJ|I\right)}\right]={\Phi }^{-1}\left[{\text{Pr}(REJ|I)}^\frac{1}{m}\right]$$

## Equation 1.2. Discriminability ${{\varvec{d}}}_{{\varvec{G}}{\varvec{I}}}^{\boldsymbol{^{\prime}}}$

$${d}_{GI}^{\prime}={\Phi }^{-1}\left[{\text{Pr}\left(REJ|I\right)}^\frac{1}{m}\right]-{\Phi }^{-1}\left[{\text{Pr}\left(REJ|I\right)}^\frac{1}{m}\frac{\text{Pr}\left(REJ|G\right)}{\text{Pr}\left(REJ|I\right)}\right]$$

In the equations, *m* denotes the line size (number of lineup members), and $(m-1)$ denotes the filler size (i.e., number of fillers). $\Phi (*)$ denotes the cumulative distribution function (cdf) of a standard normal distribution, and ${\Phi }^{-1}(*)$ denotes its inverse function (i.e., calculating quantiles of a standard normal distribution). The derivations of these equations are included in Appendix C.

## Implications of the mSDT model

Following the above introduction to the mSDT model, we highlight two key differences between the mSDT model and the classic uSDT model and discuss their implications.

### Underlying distributions

The first difference lies in the underlying decision variable distributions. The classic uSDT model addresses recognition tasks that present one *individual* recognition item for each decision. Accordingly, the model assumes a *univariate* distribution for signals of individual recognition items (either old or new). By contrast, the mSDT model addresses lineup tasks that present *multiple* recognition items for each decision. Because the decision variable involves signals from multiple lineup members, a univariate distribution may misrepresent the lineup signal distribution and fail to capture the relations among lineup members (see the discussion in the supplementary materials). Thus, the mSDT model employs a *multivariate* approach, modeling the joint distributions of lineup signals in a multivariate decision space.

By modeling the culprit-present and culprit-absent lineup signals in a multivariate space, the mSDT model goes beyond simply considering the mean difference between guilty and innocent suspects. More importantly, it captures the relations among lineup members. This ability stems from the fact that multivariate joint distributions consider these signals simultaneously within the same distribution. Visually, the suspect and filler signals within a same lineup are represented as one unified distribution (see Figs. 4, 5 and 6); mathematically, this joint distribution considers both the variances and covariances among these signals (see the section “Alternative Distribution Assumptions”). This approach, in turn, prompts important questions on the suspect–filler relations and how to best define witness discriminability (see the section “The Role of Fillers”). The use of multivariate distributions in mSDT thus offers a comprehensive and nuanced approach for modeling eyewitness identification decisions.

We conducted a simulation study to compare the accuracy in estimating model parameters when using equations derived from the mSDT model versus those from the classic uSDT model. The results showed that mSDT yielded more accurate estimates than uSDT. The convergence of the mSDT estimates to the parameters used for the simulations suggests that the mSDT model may present a more accurate framework for lineup identification tasks. Full details of the simulation are available in the supplementary materials.

Multivariate applications of SDT have significantly contributed to cognitive research (Ashby, 1992; Macmillan & Creelman, 2005). For instance, the General Recognition Theory employs multidimensional distributions to examine how individuals recognize different perceptual dimensions of the same item, such as judging both age and gender of the same face (e.g., Ashby & Gott, 1988; Ashby & Perrin, 1988; Ashby & Soto, 2015). Additionally, multivariate SDT has been applied to understand the psychological mechanisms underlying source monitoring (e.g., DeCarlo, 2003) and remember-know judgments (e.g., Rotello et al., 2004, 2006). This established body of research underscores the power of multivariate approaches in capturing the complexities of various recognition tasks, providing a strong basis for applying multivariate SDT to lineup tasks.

### Decision rules

The difference in the underlying decision variable distributions between uSDT and mSDT also relates to another key difference—flexibility in decision rules. As the classic uSDT relies on univariate distributions, it typically assumes that decision makers use a simple *linear* decision rule for their decisions (Green & Swets, 1966). This means that a single criterion is placed on the univariate continuum of the decision variable. If an observed signal exceeds the criterion, the item is identified as “old”; otherwise, it is identified as “new.”

In a rating experiment, decision makers provide confidence ratings instead of the “old-new” binary responses (or confidence ratings following the “old-new” responses). These confidence ratings are assumed to follow the same linear decision rule, making them interchangeable with an “old-new” response criterion (Macmillan & Creelman, 2005). Importantly, this “uniform decision rule” assumption facilitates the interchangeability between confidence ratings and binary decision criteria, which underpins the use of confidence rating data to generate ROC curves (e.g., Weidemann & Kahana, 2016).

In essence, when researchers use confidence ratings to generate ROC curves, it is implicitly assumed that decision makers apply the same type of decision rules (e.g., a linear rule) to both confidence ratings and “old-new” binary responses. Thus, various confidence ratings are equivalent to potential criteria people would use in making “old-new” responses. Visually, the decision criteria for confidence ratings are aligned *in parallel* with the decision criteria for binary responses along the univariate decision variable axis. By plotting response rates from different confidence levels, which are interchangeable with varying binary decision criteria, ROC curves can thus account for different decision criteria. Indeed, recognition experiments showed that confidence data and instruction data generally fall on the same ROC curve, supporting the interchangeability between confidence ratings and binary decision criteria under the classic uSDT model (e.g., Dube & Rotello, 2012; Koen & Yonelinas, 2011).

On the other hand, because of its expanded multivariate decision space, the mSDT model allows for more sophisticated decision rules that extend beyond the simple linear rule. As shown in Figs. 5, 6 and 7, the MAX decision rule—which partitions the decision space into three regions that correspond to the three types of eyewitness responses—is *not linear*. Such nonlinear decision rules suggest that it may not be appropriate to compress lineup signal distributions onto a univariate decision space; doing so may result in the loss of important information, such as the nonlinearity of decision rules.

The complexity and flexibility inherent in multivariate decision rules further necessitate considerations of not only SDT model parameters (e.g., discriminability and decision criterion) but also *witness decision rules*. Indeed, witnesses could employ many different decision rules in such a multivariate decision space. As will be discussed, many debates about proper eyewitness SDT models center around various decision rules that witnesses may adopt, rather than assuming different underlying memory distributions. In other words, the debate is more about which lineup procedure encourages witnesses to adopt optimal decision rules, rather than enhancing discriminability between guilty and innocent suspects.

Most importantly, the flexibility of decision rules permitted by the multivariate decision space raises an important question: Do witnesses apply the same type of rules for lineup identification decisions and subsequent confidence ratings? If not, the “uniform decision rule” assumption might be compromised, invalidating the equivalence between confidence ratings and identification decision criteria. This potential violation has important implications for eyewitness ROC analysis. We expand on this point in more detail later.

## Alternative model assumptions

The assumptions used in the above model are highly useful for model development—yet they may oversimplify the complexities inherent in lineup data. Below, we discuss alternative model assumptions that could accommodate more sophisticated considerations. We first explore possible modifications to the underlying signal distributions. We then consider possible modifications to the decision rules.

### Alternative distribution assumptions

We begin by examining potential variations in the underlying distributions of lineup member signals. These assumptions directly impact the shapes of the distributions.

#### Unequal variances

The original mSDT model assumes equal variance across all lineup signals. However, a substantial body of literature has shown that empirical data from recognition tasks frequently produce asymmetrical ROC curves (Yonelinas & Parks, 2007). This robust finding challenges the predictions derived from the equal-variance assumption, suggesting that such an assumption is often violated in practice. Although the specific factors contributing to the asymmetry of empirical ROC curves remain a topic of ongoing debate (Yonelinas, 2024), one plausible explanation is that the signal distributions may have different variances (Wixted, 2007).

Recognizing these well-documented findings, an important modification to the model is to allow for unequal variances across different lineup members. This modification would account for potential differences in variability among lineup member signals, particularly between the guilty suspect and other lineup members. By incorporating these potential differences, the model would offer a more accurate representation of witness decision-making processes. We depict a visual representation of these distributions with unequal variances in Appendix D.

#### Correlated lineup member signals

The original model assumes independence among lineup member signals. This assumption, though, may not hold because lineup members often share some common features (Shen et al., 2023; Wixted et al., 2018). Specifically, the correlations among lineup member signals would increase as they become more similar to each other. In other words, whereas one witness may have comparably strong signals for all lineup members, another witness may have weak signals for all lineup members.

In terms of model assumptions, lineup member signals would not follow independent normal distributions any further. Instead, we could assume that their signals follow a multivariate normal distribution, which allows for nonzero correlations among signals from the same witness. For culprit-absent lineups,

$${\left[\begin{array}{c}ss\\ \begin{array}{c}f{s}_{1}\\ f{s}_{2}\\ \begin{array}{c}\vdots \\ f{s}_{j}\end{array}\end{array}\end{array}\right]}_{i} \overset{\mathrm{i.i.d.}}{\sim} \; \mathcal{MVN}\left(\varvec{\mu_{CA} } =\left[\begin{array}{c}0\\ 0\\ \begin{array}{c}0\\ \vdots \\ 0\end{array}\end{array}\right], \; \Sigma =\left[\begin{array}{ccc}1& \rho & \begin{array}{ccc}\rho & \dots & \rho \end{array}\\ \rho & 1& \begin{array}{ccc}\rho & \dots & \rho \end{array}\\ \begin{array}{c}\rho \\ \vdots \\ \rho \end{array}& \begin{array}{c}\rho \\ \vdots \\ \rho \end{array}& \begin{array}{c}\begin{array}{ccc}1& \dots & \rho \end{array}\\ \begin{array}{ccc}\vdots & \ddots & \vdots \end{array}\\ \begin{array}{ccc}\rho & \dots & 1\end{array}\end{array}\end{array}\right]\right)$$

For culprit-present lineups,

$${\left[\begin{array}{c}ss\\ \begin{array}{c}f{s}_{1}\\ f{s}_{2}\\ \begin{array}{c}\vdots \\ f{s}_{j}\end{array}\end{array}\end{array}\right]}_{i} \overset{\mathrm{i.i.d.}}{\sim} \; \mathcal{MVN}\left(\varvec{\mu_{CP}} = \left[\begin{array}{c}{d}_{GI}^{\prime}\\ 0\\ \begin{array}{c}0\\ \vdots \\ 0\end{array}\end{array}\right], \; \Sigma =\left[\begin{array}{ccc}1& \rho & \begin{array}{ccc}\rho & \dots & \rho \end{array}\\ \rho & 1& \begin{array}{ccc}\rho & \dots & \rho \end{array}\\ \begin{array}{c}\rho \\ \vdots \\ \rho \end{array}& \begin{array}{c}\rho \\ \vdots \\ \rho \end{array}& \begin{array}{c}\begin{array}{ccc}1& \dots & \rho \end{array}\\ \begin{array}{ccc}\vdots & \ddots & \vdots \end{array}\\ \begin{array}{ccc}\rho & \dots & 1\end{array}\end{array}\end{array}\right]\right)$$

In the above assumptions, *ρ* denotes the correlations among lineup member signals. ${d}_{GI}^{\prime}$ denotes the mean difference between guilty suspect and innocent suspect distributions.

**Fig. 8:**
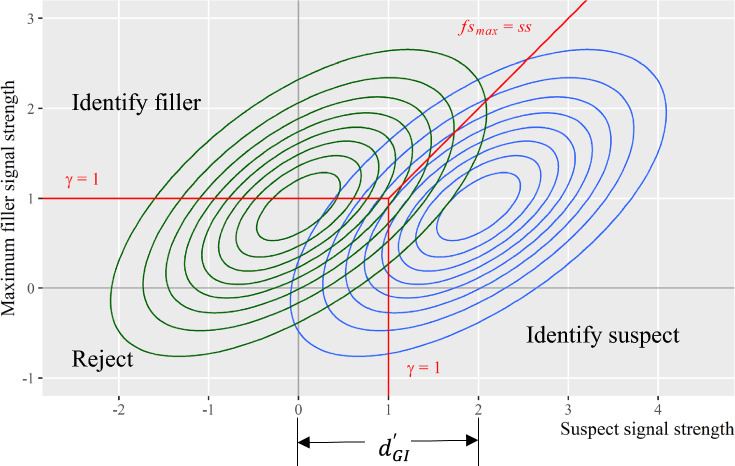
Joint Distributions Assuming Correlated Signals among Lineup Members

Note that the max filler distribution will change if one assumes nonzero correlations among the signals (see Arellano-Valle & Genton, 2008). Figure 8 displays the contour plots of the joint distributions when assuming correlated signals (specifically, $\rho =0.5$ and ${d}_{GI}^{\prime}=2$). Interested readers can refer to Appendix E for the derivations of the joint pdfs.

### Alternative decision rules

The mSDT model highlights the importance of witness decision rules. Indeed, the multivariate decision space enables witnesses to use more sophisticated decision rules beyond the simple linear rule. Below, we explore some potential variations to witness decision rules.

#### Criterion shift

The original mSDT model assumes the same decision criterion for both culprit-present and culprit-absent lineups. However, because lineup tasks are between-subjects rather than within-subjects, witnesses may not use the same decision criterion across culprit-present and culprit-absent lineups (Smith et al., 2018). Put differently, a criterion shift may happen between culprit-present lineups and culprit-absent lineups even when the same lineup procedure is used. Thus, a potential modification to the model is to account for the possibility that witnesses may use different decision criterion across culprit-present and culprit-absent lineups.

#### DIFF decision rule

The original mSDT model assumes the MAX decision rule. Given the multivariate space, other decision rules are possible. For example, the WITNESS model discusses the DIFF decision rule, in which a lineup member will be identified if it has the strongest signal and if the difference between its signal and the next-best signal exceeds a decision criterion (Clark, 2003).

**Fig. 9:**
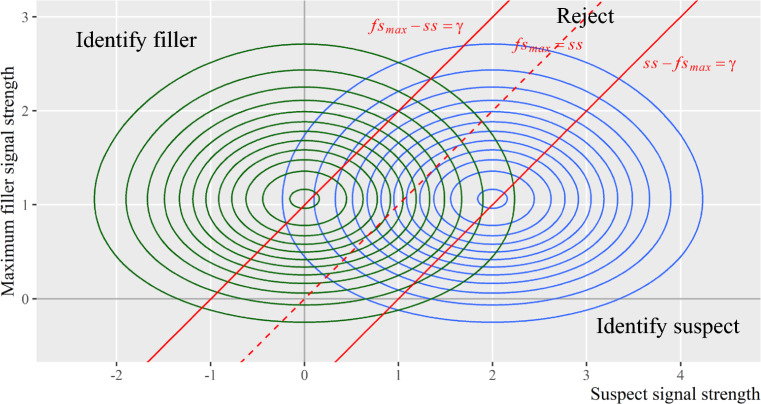
Map the DIFF Decision Rule. *Note:* The red lines depict the DIFF decision rule. The suspect signal is assumed to be either the best or the next-best signal in a lineup

As shown in Fig. 9, the DIFF decision rule can be mapped onto the multivariate decision space when the suspect signal is either the best or the next-best signal. When the suspect signal exceeds the max filler signals (i.e., suspect signal is the best), witnesses will identify the suspect if the difference between suspect and max filler signals surpasses the decision criterion (to the right of the red solid line, $ss-f{s}_{max}=\gamma$). When the suspect signal does not exceed the max filler signal, likewise, witnesses will identify the filler if the magnitude of the difference surpasses the decision criterion (to the left of the red solid line, $f{s}_{max}-ss=\gamma$). The area in between shows the situation in which the difference does not exceed the decision criterion, which would then lead to a lineup rejection.

#### Integration decision rule

**Fig. 10:**
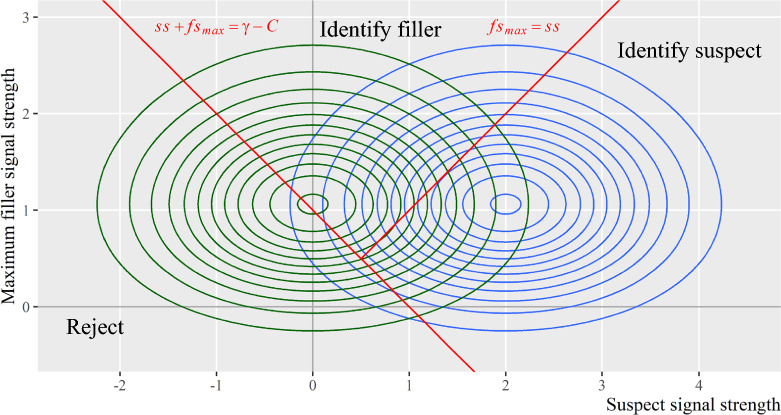
Map the Integration Decision Rule*. Note:* The red lines depict the integration decision rule. *C* stands for the sum of all filler memory signals except the max filler

The compound signal detection model discusses the integration decision rule, in which a lineup member will be identified when it has the strongest signal and when the sum of all lineup members exceeds a decision criterion (Duncan, 2006). As shown in Fig. 10, the integration decision rule can be mapped on the multivariate decision space. Witnesses will reject the lineup when the sum of the suspect and max filler signals does not exceed the difference between the decision criterion and the sum of other fillers (to the left of the red solid line $ss+f{s}_{max}=\gamma -C$; *C* stands for the sum of other filler memory signals). Otherwise, witnesses will identify the lineup member with the strongest signal. Here the sum of other filler signals is approximated as a constant. To depict the integration rule more precisely, the decision space needs to be expanded to incorporate the sum of other filler signals to account for its stochasticity.

#### Ensemble decision rule

**Fig. 11:**
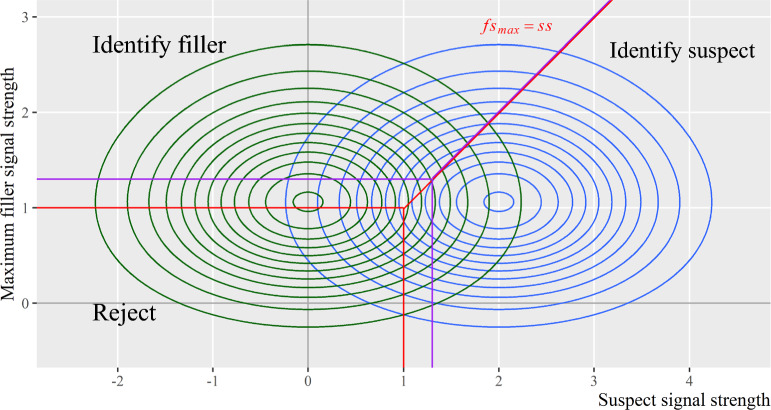
Map the Ensemble Model Decision Rule*. Note:* The red lines depict witnesses’ decision criterion in culprit-absent lineups. The purple lines depict witnesses’ decision criterion in culprit-present lineups

The Ensemble model treats the difference between a lineup member’s signal and the average signal of all lineup members as the decision variable and applies the MAX decision rule to the difference scores (Wixted et al., 2018). As shown in Appendix F, these assumptions can be approximated as applying the MAX decision rule to the original signals with a criterion shift between culprit-present and culprit-absent lineups. Figure 11 presents such assumptions onto the decision space of the original signals. Specifically, applying the MAX decision rule to the difference scores implies a more conservative decision criterion for culprit-present lineups than culprit-absent lineups.

### Model complexity and over-parameterization

We would like to emphasize that the alternative assumptions discussed above are speculative considerations rather than established principles. Adopting all these assumptions could lead to an overly complicated model, posing challenges for model estimation and interpretation (Schoenenberger et al., 2017). It is crucial for future research to evaluate these assumptions and develop a parsimonious model—one that achieves simplicity without compromising the ability to capture the essence of empirical data (Forster, 2000; Schoenenberger et al., 2017).

## Further implications of the mSDT model

Theory development is essential for uncovering the structures and mechanisms of real-world phenomena (Hintzman, 1991). Mathematical modeling, among others, stands as a profound form of theory development (Luce, 1995). Yet, as noted by the National Research Council (2014), “this strong scientific foundation remains insufficient for understanding the strengths and limitations of eyewitness identification procedures in the field” (p. 113). Through a mathematical modeling and visualization approach, the mSDT model provides an important theoretical framework for understanding and analyzing eyewitness identification decisions, thereby building a strong foundation for eyewitness research. Expanding upon our earlier discussion, we delve deeper into the implications of the mSDT model.

### The role of decision rules and eyewitness ROC analysis

The multivariate decision space inherent in mSDT illuminates the role of decision rules. Unlike the default linear rule in the classic uSDT model, witnesses may adopt more complex decision rules in a lineup task. As discussed, witnesses could potentially employ many different decision rules, which shed light on the application of ROC analysis to eyewitness decisions.

Let us return to the long-standing debate in eyewitness literature regarding how to construct the eyewitness ROC curves given the 3 × 2 data structure (Lampinen, 2016; Wells et al., 2015). A common practice is to only use suspect identification rates with various confidence levels to create “partial” suspect-only ROC curves (e.g., Wixted & Mickes, 2012). While it remains debatable whether such “partial” ROC curves reflect witness discriminability or investigator discriminability (Smith et al., 2020; Yang & Smith, 2023), the mSDT model identifies key issues in the current eyewitness ROC practices that might hinder disentangling discriminability from decision criteria.

**Fig. 12:**
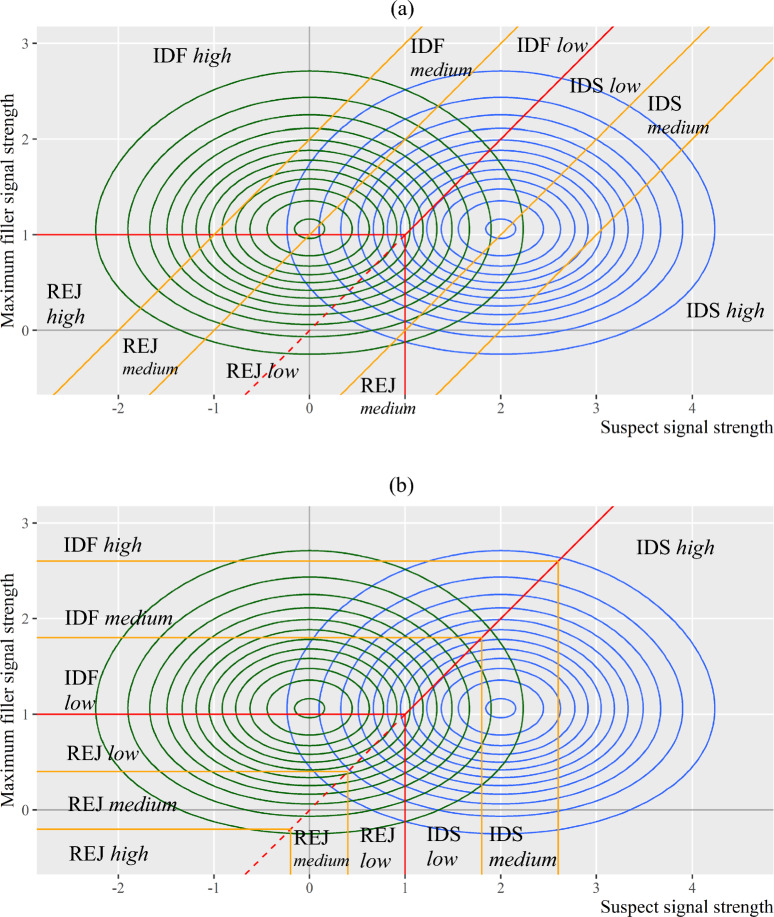
Hypothetical Decision Rules for Confidence Ratings in Lineup Tasks. *Note*: The red lines depict the decision rule for identification decisions. The orange lines depict the decision rule for confidence ratings. **a** Difference decision rule for confidence ratings. **b** Absolute decision rule for confidence ratings

First and foremost, the flexibility in the multivariate decision rules opens up the possibility that witnesses may use different types of rules for identification decisions and confidence decisions. Figure 12 depicts two examples of decision rules that witnesses might use for confidence ratings, shown as orange lines. The top panel shows that witnesses rate their confidence based on the *difference* between lineup member signals, and the bottom panel shows that witnesses rate their confidence based on the *absolute* strength of lineup member signals.

Consider the top panel in Fig. 12. If witnesses rely on the differences between lineup member signals for their confidence ratings, it is evident that these confidence ratings follow a distinct type of decision rules (shown as orange lines) compared to the rules used for identification decisions (shown as red lines). Put differently, when witnesses adjust criteria for identification decisions, these new criteria will not map onto the criteria used for confidence ratings, implying that confidence ratings do not necessarily represent potential criteria for identification decisions.

In fact, eyewitness research has provided evidence of divergence between identification and confidence decision rules in lineup tasks. For example, unlike recognition tasks, confidence data and instruction data from lineup tasks did not map onto the same ROC curves (Mickes et al., 2017). In addition, a recent study found that witnesses may rely on absolute judgments for identification decisions but relative judgments for confidence ratings (Smith et al., 2024), further indicating a divergence between identification and confidence decision rules.

In addition, the above analysis suggests that confidence ratings may not follow the same type of decision rules as identification decisions. This still relies on the assumption that confidence ratings are based on the same decision variable as identification decisions—namely the signal strength of lineup members. However, recent developments in metacognition research suggest that confidence ratings may not use the same source of information as recognition decisions (Fleming, 2024; Fleming & Daw, 2017). Instead, they could be influenced by other factors, such as heuristic cues and post-decision processes (e.g., Alter & Oppenheimer, 2009; Pleskac & Busemeyer, 2010). These discrepancies between confidence ratings and identification decisions thus undermine the theoretical basis for using confidence data to construct eyewitness ROC curves. In essence, the confidence ROC points may not accurately reflect witness responses across varying *identification* decision criteria, thereby failing to account for these criteria.

A second challenge in constructing eyewitness ROC curves stems from the nonlinearity of the decision rules. The bottom panel in Fig. 12, which assumes witnesses base their confidence ratings on absolute signal strengths, provides a hypothetical example in which confidence ratings could potentially substitute for identification criteria. Even in such instances, creating eyewitness ROC curves that are criterion-free may not be feasible due to the nonlinear nature of the decision rules. Without a linear decision rule, it may not be possible to map the multivariate distributions onto a univariate space, which is essential for developing ROC curves for eyewitness identification tasks.

A third issue in eyewitness ROC analysis involves potential confounding between decision rules and discriminability. The mSDT model reveals that multiple types of decision rules are possible in a multivariate decision space. In such situations, even when the underlying signal distributions remain identical, employing different decision rules could still lead to different ROC curves. In other words, differences in ROC curves may arise from different decision rules rather than changes in the latent signal distributions. For example, witnesses may employ a MAX decision rule in simultaneous lineups but opt for a linear decision rule in sequential lineups (Palmer & Brewer, 2012). Even if researchers were able to overcome the aforementioned challenges and successfully construct eyewitness ROC curves, it remains unclear whether differences in these ROC curves result from different decision rules or different latent discriminability between guilty and innocent suspect signals.

Given these challenges, we believe that the current lineup ROC practices may not accurately reflect witness latent discriminability. Whereas one could possibly use lineup data to construct *investigator* ROC curves that reflect investigator classification tasks (Smith et al., 2020; Yang & Smith, 2023), the challenges suggested by the mSDT model post questions on how to create ROC curves that reflect *witness* identification tasks. Future research is needed to address these issues and develop innovative methods to construct eyewitness ROC curves that effectively capture witness decision making.

### The role of fillers

Not only does the mSDT model highlight the importance of witness decision rules, it also takes lineup fillers into consideration. The inclusion of lineup fillers has important implications for understanding eyewitness identification decisions.

#### Within-lineup and between-lineup comparisons

The distributions in Fig. 7 primarily compare culprit-present and culprit-absent lineups. The mSDT model, however, also enables comparisons within either culprit-present lineups or culprit-absent lineups. This idea is inspired by the *m*-alternative forced choice (mAFC) paradigm (Green & Swets, 1966), which likely depends on underlying signal distributions similar to culprit-present lineups.

**Fig. 13:**
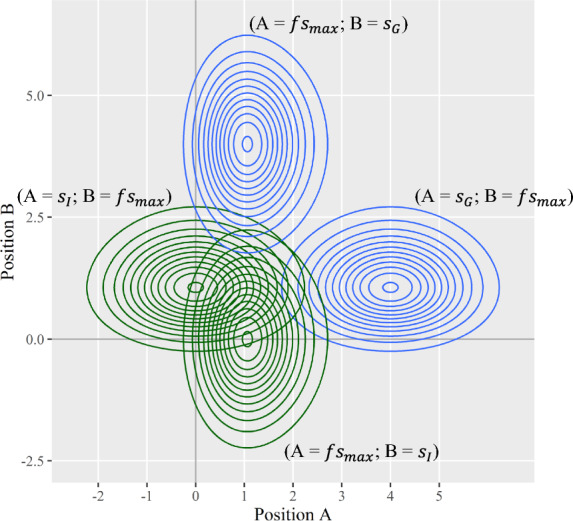
Decision Space For Lineup Tasks Assuming Alternating Positions. *Note:* The blue contours depict culprit-present lineups. The green contours depict culprit-absent lineups

Figure 13 shows a configuration of mSDT that enables within-lineup comparisons. In this setup, we assume that the suspect and the max filler can interchangeably occupy any two random positions, A and B. Accordingly, distributions of lineup member signals can be depicted as four distributions. The two blue contours depict culprit-present lineups, with the guilty suspect and max filler alternating positions. The two green contours depict culprit-absent lineups, with the innocent suspect and max filler alternating positions. Analyzing the two blue contours would then shed light on witness behaviors within culprit-present lineups, whereas analyzing the two green contours would shed light on witness behaviors within culprit-absent lineups.

#### How to define witness discriminability?

The distinction between within-lineup and between-lineup comparisons, along with the multivariate presentations, raises fundamental questions about witness discriminability. In the model assumptions, we define “discriminability” specifically as the mean difference between guilty and innocent suspect signals, ${d}_{GI}^{\prime}$, which is also visualized in Fig. 7 as the marginal mean difference between culprit-present and culprit-absent distributions.

However, Fig. 13 reveals that other mean differences exist in this multivariate decision space. For example, the within-lineup comparisons highlight the marginal mean difference between suspect and max filler signals. In addition, the Euclidean mean difference between the joint distributions may not equate to the marginal mean differences (Casell & Berger, 2002). Furthermore, if assuming witnesses use *linear* decision rules, such as the difference decision rule used in mAFC tasks, these multivariate distributions could be projected onto a univariate (*A-B*) axis, yielding mean differences between the transformed univariate distributions. Given various possible ways to examine these distributional differences, how would they influence the analysis of witness responses? How should researchers define witness discriminability? Which measure of discriminability should be used to quantify performance? Again, the mSDT model raises important new questions that await future research and discussion.

### Distinguish between lineup fairness and filler similarity

Eyewitness researchers often use “lineup fairness” and “filler similarity” interchangeably (e.g., Colloff et al., 2021; Lee et al., 2022). But are these two concepts actually equivalent? Clarifying these concepts is important for understanding and improving the choice of lineup fillers, which can significantly impact eyewitness performance (Fitzgerald et al., 2013). The mSDT model helps to elucidate the distinction between “lineup fairness” and “filler similarity.”

#### Lineup fairness

Lineup fillers serve the purpose of protecting innocent suspects from mistaken identifications (Wells, 1993; Wells et al., 1979). Accordingly, a “fair” (or “unbiased”) lineup is defined to be a lineup in which the suspect does not stand out (Brigham et al., 1990; Well et al., 2020). Translating into SDT language, a fair lineup implies that the suspect and a filler should elicit similar signal distributions when presented to people who have no prior exposure to any of the lineup members. Such situations occur when witnesses view culprit-absent lineups (Quigley-McBride & Wells, 2021) or when mock witnesses view either culprit-present or culprit-absent lineups (the mock witness paradigm; Doob & Kirshenbaum, 1973). In these cases, the suspect would have an equal chance of being identified as any of the fillers (see Appendix B for the derivation). As will be discussed below, this definition of “lineup fairness” clearly differs from the concept of “filler similarity.”

#### Filler similarity

When two faces exhibit greater similarity, they share more features in common, resulting in an increased correlation between their memory signals (Luus & Wells, 1991; Shen et al., 2023; Wells et al., 1993). Although there can be variations in similarity values among different pairs of lineup members, we consider a simplified situation in which all possible pairs of lineup members have the same level of similarity (i.e., a constant correlation among all possible pairs). For mock witnesses or witnesses viewing a culprit-absent lineup, if all lineup members elicit similar signal distributions, the similarity among lineup members should not influence lineup fairness. This is because lineup fairness concerns the *marginal* distributions of lineup member signal distributions—particularly their means—rather than the correlations among these signals.

**Fig. 14:**
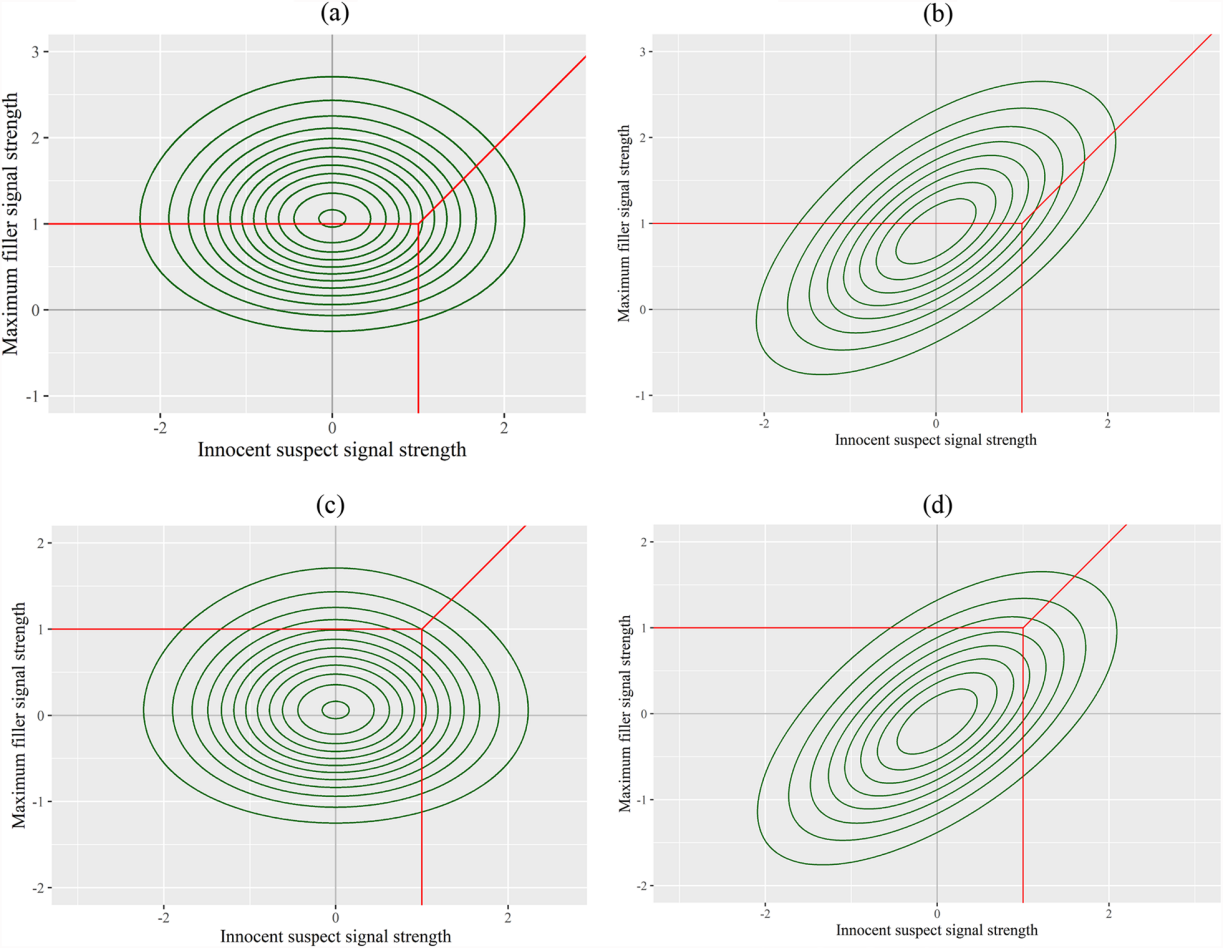
Lineup Fairness and Filler Similarity*. Note:* The red lines represent the decision criterion, which is assumed to be constant across all conditions. **a** A fair lineup with dissimilar members. **b** A fair lineup with similar members. **c** An unfair lineup with dissimilar members. **d** An unfair lineup with similar members

As shown in the top panels of Fig. 14, both low similarity and high similarity lineups could be fair as long as all lineup members elicit similar marginal distributions. Under such circumstances, the suspect would have an equal chance of being identified as any of the fillers. However, a lineup becomes unfair if the marginal distribution of the suspect is not the same as those of fillers. As shown in the bottom panels of Fig. 14, if the suspect elicits stronger signals than do fillers (i.e., ${\mu }_{I}>{\mu }_{F}$), then the suspect will have a higher chance of being identified than fillers. Such a case could happen for both low similarity and high similarity lineups.

### Implications, limitations, and future directions

To summarize, the mSDT model offers important theoretical advances for eyewitness research and sheds light on the current debates around eyewitness ROC analysis. By modeling max filler distributions and multivariate joint distributions, mSDT effectively accounts for the role of lineup fillers, providing a more nuanced understanding of how lineup members interactively impact witness decision-making.

The multivariate decision space in mSDT accommodates sophisticated decision rules, which partition the joint distributions into three distinct segments that correspond to different eyewitness responses. As such, the model accounts for the 3 × 2 structure inherent in lineup data. The potential complexity involved in witness decision rules further challenges the theoretical basis for using confidence ratings to create eyewitness ROC curves. Indeed, witnesses may not use the same type of rules or the same source of information for identification decisions and subsequent confidence ratings. As a result, varying confidence ratings may not correspond to potential identification criteria, complicating the construction of ROC curves that can effectively disentangle witness discriminability from identification criteria.

In addition, the mSDT model offers insights into the effects of various lineup factors. For example, the above discussion distinguishes between lineup fairness and filler similarity. Yet, a remaining question is how filler similarity influences eyewitness responses within fair lineups (e.g., Colloff et al., 2021). Using mSDT, researchers can examine how lineup signal distributions change as a function of filler similarity. When lineup members become more similar to each other, not only will the correlation increase, but their marginal means will tend to converge; in other words, the mean of the joint distribution will move toward the line of equality (${fs}_{max}=ss$). By examining how filler similarity influences lineup signal distributions, the mSDT model could help explain how filler similarity affects witness performance and inform optimal ways to select lineup fillers.

Another important question in eyewitness research concerns how lineup size affects eyewitness responses (Juncu & Fitzgerald, 2021). The mSDT model could help to address this question by examining max filler distributions. As discussed earlier, larger lineup sizes increase the mean and decrease the variance of max filler distributions. These shifts in max filler distributions consequently affect the joint distributions. Thus, the mSDT model provides a theoretical basis for analyzing the effect of lineup size on witness responses.

Beyond the theoretical contributions, mSDT casts important implications for real-world lineup practices. For example, the model’s ability to analyze sophisticated decision rules suggests that lineup practices could be designed to encourage witnesses to adopt more optimal strategies, potentially reducing errors. The explicit modeling of relations among lineup members could inform the selection of lineup fillers, helping to determine the ideal level of similarity among suspect and fillers. In addition, the model’s framework for understanding max filler distributions could inform decisions on the optimal lineup size, striking a balance between including enough fillers to reduce mistaken identifications and avoid the risk of losing accurate identifications in larger lineups.

Whereas we are enthusiastic about the potential of the mSDT model, we acknowledge that it remains in its initial stage and has not been fully developed. Specifically, the current format of the model is based on a set of constrained assumptions, which may limit its ability to explain data. Although we have considered alternative assumptions, these remain largely speculative and need empirical testing and evaluation to refine the model into a more powerful and parsimonious form.

Furthermore, the mSDT model raises many new questions rather than providing answers. For example, the model distinguishes between underlying decision variable distributions and decision rules for witness lineup tasks. This distinction prompts questions such as: What is the appropriate distribution to assume for the decision variable? What decision rules do witnesses actually use in practice? What are the optimal decision rules that witnesses should follow?

This distinction further raises questions regarding the effects of various lineup variables: Does a particular factor influence the underlying decision variable distributions, witness decision rules, or both? Grasping these effects is crucial for understanding witness decision making and improving lineup procedures. At the same time, it also poses methodological challenges in designing experiments that can disentangle these two components.

Additionally, the complexity involved in the multivariate decision rules raises questions on the best methods for creating ROC curves that accurately capture witness discriminability. Addressing these questions requires substantial research and development, foreseeing a considerable journey ahead in refining and applying the mSDT framework.

## Conclusion

This paper proposes an mSDT model to better understand how witnesses use their memory to make lineup decisions. The model addresses a major challenge in eyewitness research: the presence of lineup fillers, which creates a decision task that deviates from the binary recognition decisions typically addressed by SDT (Lampinen, 2016; Wells et al., 2015). Related to this challenge, previous eyewitness models generally present lineup tasks in a univariate decision space, neglecting to fully integrate filler signals. The mSDT model addresses this issue by incorporating the joint distributions of suspect and filler signals and presenting these distributions and witness decision rules in a multivariate decision space. The visual format of the model is particularly important because it allows for incorporating suspect–filler relations and complex decision rules.

The mSDT model not only sheds light on the debates around eyewitness SDT and ROC analysis, but also helps to scientifically understand eyewitness identification decisions, with implications for lineup construction and application to eyewitness data. The model additionally has promise to advance knowledge across different fields, such as the basic memory and perception research when multiple signal detection items are involved. The mSDT model therefore provides a useful theoretical framework for understanding and analyzing eyewitness identification decisions, strengthening the theoretical foundation for eyewitness research.

## Supplementary Information

Additional file 1.

## Figures and Tables

**Fig. A1:**
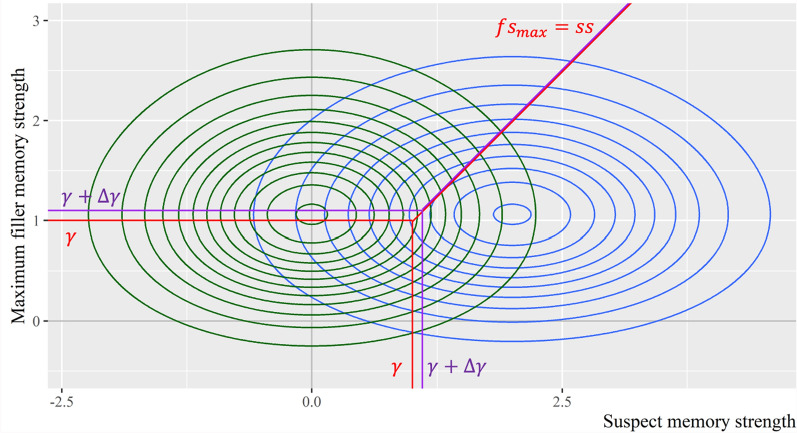
Assume Unequal Variances and a Criterion Shift between Culprit-Present and Culprit-Absent Lineups. *Note*: The blue contours are more spread out than the green contours in the *x*-axis direction because the guilty suspect distribution is assumed to have a larger variance than the innocent suspect distribution. The red lines depict witnesses’ decision criterion in culprit-absent lineups, and the purple lines depict witnesses’ decision criterion in culprit-present lineups

## Data Availability

The R code used for the analysis is available at osf.io/n2zbc/. The manuscript does not include any empirical data.

## Appendix A: Max filler probability distribution

The MAX decision rule implies that witnesses' decision process involves the memory signal of the filler that has the strongest memory strength (“max filler”). Therefore, multiple filler signals in a lineup can be reduced to one—the max filler signal. We can derive the probability density function of the max filler distribution.

In the model, we assume that a lineup’s filler signals are independent random draws from a standard normal distribution,

$${fs}_{j} \overset{\mathrm{i.i.d.}}{\sim} \mathcal N(0, 1)$$

Therefore, the cumulative distribution function (cdf) of the max filler signal follows,

$$F_{{fs_{max} }} \left( y \right) = \Pr\left( {fs_{max} < y} \right) = \Pr\left( {\mathrm{all}\space fs_{j} < y} \right) = \left[ {\Pr\left( {fs_{j} < y} \right)} \right]^{m - 1} = \left[ {{\Phi }\left( y \right)} \right]^{m - 1}$$

Here *m* denotes the lineup size (i.e., number of lineup members). Correspondingly, $(m-1)$ denotes the filler size (i.e., number of fillers). $\Phi \left(*\right)$ denotes the cdf of a standard normal distribution. From the cdf, one can derive the probability density function (pdf) of the max filler signal,

$$f_{{fs_{max} }} \left( y \right) = \frac{{\text{d}F_{{fs_{max} }} \left( y \right)}}{\text{d}y} = \frac{{\text{d}\left[ {{\Phi }\left( y \right)} \right]^{m - 1} }}{\text{d}y} = \left( {m - 1} \right)\left[ {{\Phi }\left( y \right)} \right]^{m - 2} \frac{{\text{d}{\Phi }\left( y \right)}}{\text{d}y} = \left( {m - 1} \right)\left[ {{\Phi }\left( y \right)} \right]^{m - 2} \phi \left( y \right)$$

Here $\phi \left(*\right)$ denotes the pdf of a standard normal distribution.

The closed-form expressions of the distribution mean and variance are not easy to derive (David & Nagaraja, 2003). Yet we were able to obtain their numeric approximations (see Table A1).

**Table A1:** Mean, Variance, and Skewness of Max Filler Distributions in Different-Sized Lineups

Lineup size	Filler size	${\mu}_{max}$	${\sigma}_{max}^{2}$	*Skewness*
2	1	0.000	1.000	0.000
3	2	0.564	0.682	0.137
4	3	0.846	0.559	0.213
5	4	1.029	0.492	0.265
6	5	1.163	0.448	0.303
12	11	1.586	0.333	0.423
20	19	1.844	0.280	0.494
30	29	2.029	0.248	0.543
40	39	2.151	0.229	0.573
50	49	2.241	0.217	0.595

## Appendix B: Derive Probabilities of Eyewitness Outcomes

With the joint distributions of suspect and filler signals, one can derive the probabilities of the three eyewitness outcomes in both culprit-present and culprit-absent lineups. Specifically, the pdf of the joint distribution of suspect and max filler signals would be,

$${f}_{ss, f{s}_{max}}\left(x, y\right)={f}_{ss}\left(x\right){f}_{f{s}_{max}}\left(y\right)$$

Unlike the order in the manuscript, we first examine culprit-absent lineups because of the relative ease in its derivations. We then turn to culprit-present lineups.

## Culprit-Absent Lineups

In culprit-absent lineups, witnesses' signal detection task can be described by a joint distribution of *innocent* suspect and max filler signals. Figure 6 in the manuscript displays this joint distribution in a contour plot. Note that for this joint distribution, the mean of the innocent suspect marginal distribution is zero. This joint distribution is partitioned into the three segments by the MAX decision rule, shown as the three red lines in Fig. 6. The volume of each segment corresponds to the probability of each of the three eyewitness outcomes in *culprit-absent* lineups.

As mentioned above, the pdf of the joint distribution is,

$$f_{{ss_{I} , fs_{\max } }} \left( {x, y} \right) = f_{{ss_{I} }} \left( x \right)f_{{fs_{\max } }} \left( y \right) = \left( {m - 1} \right)\phi \left( x \right)\phi \left( y \right)\left[ {\Phi \left( y \right)} \right]^{m - 2}$$

From the above model, one can derive the probabilities of the three eyewitness outcomes in culprit-absent lineups. One can derive these probabilities using two different approaches. The first approach uses integral calculus to integrate the pdf of the joint distribution of innocent suspect and max filler signals over each of the three regions divided by the MAX decision rule.

$$\Pr \left( {REJ|I} \right) = \mathop \int \limits_{ - \infty }^{\gamma } \mathop \int \limits_{ - \infty }^{\gamma } f_{{ss_{I} }} \left( x \right)f_{{fs_{\max } }} \left( y \right)\text{d}x \space \text{d}y = \mathop \int \limits_{ - \infty }^{\gamma } f_{{ss_{I} }} \left( x \right)\text{d}x\mathop \int \limits_{ - \infty }^{\gamma } f_{{fs_{\max } }} \left( y \right)\text{d}y = \mathop \int \limits_{ - \infty }^{\gamma } \phi \left( x \right)\text{d}x\mathop \int \limits_{ - \infty }^{\gamma } \left( {m - 1} \right)\left[ {\Phi \left( y \right)} \right]^{m - 2} \phi \left( y \right)\text{d}y = \Phi \left( \gamma \right) \cdot \Phi \left( \gamma \right)^{m - 1} = \Phi \left( \gamma \right)^{m}$$

$$\Pr \left( {IDS|I} \right) = \mathop \int \limits_{\gamma }^{ + \infty } \mathop \int \limits_{ - \infty }^{x} f_{{ss_{I} }} \left( x \right)f_{{fs_{\max } }} \left( y \right)\text{d}y \space \text{d}x = \mathop \int \limits_{\gamma }^{ + \infty } f_{{ss_{I} }} \left( x \right)\mathop \int \limits_{ - \infty }^{x} \text{d}F_{{fs_{\max } }} \left( y \right)\text{d}x = \mathop \int \limits_{\gamma }^{ + \infty } f_{{ss_{I} }} \left( x \right)\Phi \left( x \right)^{m - 1} \text{d}x = \mathop \int \limits_{\gamma }^{ + \infty } \phi \left( x \right)\Phi \left( x \right)^{m - 1} \text{d}x = \mathop \int \limits_{\gamma }^{ + \infty } \Phi \left( x \right)^{m - 1} \text{d}\Phi \left( x \right) = \frac{1}{m}\left[ {1 - \Phi \left( \gamma \right)^{m} } \right]$$

$$\Pr \left( {IDF|I} \right) = \mathop \int \limits_{\gamma }^{ + \infty } \mathop \int \limits_{ -\infty }^{y } f_{ss_{I}} \left(x\right)f_{fs_{\max}} \left(y\right) \text{d}x \; \text{d}y = \mathop \int \limits_{\gamma}^{ +\infty } f_{fs_{\max}} \left(y \right)\mathop \int \limits_{ -\infty }^{y} \phi \left(x\right) \text{d}x \; \text{d}y = \mathop \int \limits_{\gamma }^{ +\infty } f_{fs_{\max} } \left( y \right)\Phi\left(y\right) \text{d}y = \mathop \int \limits_{\gamma }^{ +\infty } \left( {m - 1} \right)\left[ {\Phi \left(y\right)} \right]^{m - 2} \phi \left(y\right) \Phi \left(y\right) \text{d}y = \mathop \int \limits_{\gamma }^{ +\infty } \left( {m - 1} \right)\left[ {\Phi \left(y\right)} \right]^{m - 1} \text{d}\Phi\left(y\right) = \frac{m - 1}{m}\left[ {1 - \Phi \left( \gamma \right)^{m} } \right]$$

In the equations, *m* denotes the line size, and $\left( {m - 1} \right)$ denotes the filler size. ${\Phi }\left( * \right)$ denotes the cdf of a standard normal distribution, and ${\Phi }^{ - 1} \left( * \right)$ denotes its inverse function (i.e., calculating quantiles of a standard normal distribution).

The second approach derives the probabilities directly from the conceptual definitions.

$$\Pr \left( {REJ{|}I} \right) = \Pr \left( {{\text{all signals}} < \gamma |I} \right) = \Pr \left( {ss < \gamma |I } \right)\Pr \left( {fs_{1} < \gamma |I} \right)\Pr \left( {fs_{2} < \gamma |I} \right) \cdots \Pr \left( {fs_{m - 1} < \gamma |I} \right) = {\Phi }\left( \gamma \right)^{m}$$

According to the mathematical constraint that all probabilities sum up to 1,

$$\Pr \left( {IDS{|}I} \right) + \Pr \left( {IDF{|}I} \right) = 1 - \Pr \left( {REJ{|}I} \right) = 1 - {\Phi }\left( \gamma \right)^{m}$$

In a fair culprit-absent lineup, all lineup members have an equal chance to be identified. This is reflected in the model assumptions that both innocent suspect and filler signals are independent random draws from the same standard normal distribution. In other words, an innocent suspect has an equal chance of being identified as any of the fillers. Accordingly,

$$\Pr \left( {IDS{|}I} \right) = \frac{1}{m}\left[ {\Pr \left( {IDS{|}I} \right) + \Pr \left( {IDF{|}I} \right)} \right] = \frac{1}{m}\left[ {1 - {\Phi }\left( \gamma \right)^{m} } \right]$$

$$\Pr \left( {IDF{|}I} \right) = \frac{m - 1}{m}\left[ {\Pr \left( {IDS{|}I} \right) + \Pr \left( {IDF{|}I} \right)} \right] = \frac{m - 1}{m}\left[ {1 - {\Phi }\left( \gamma \right)^{m} } \right]$$

Which lead to the same results as the calculus derivations.

## Culprit-Present Lineups

In culprit-present lineups, witnesses' signal detection task can be described by a joint distribution of *guilty* suspect and max filler signals. Figure 5 in the manuscript displays this joint distribution in a contour plot. This joint distribution is also partitioned into the three segments by the MAX decision rule, shown as the three red lines in Fig. 5. The volume of each segment corresponds to the probability of each of the three eyewitness outcomes in *culprit-present* lineups.

As mentioned above, the pdf of the joint distribution is,

$$f_{{ss_{G} , fs_{max} }} \left( {x, y} \right) = f_{{ss_{G} }} \left( x \right)f_{{fs_{max} }} \left( y \right) = \left( {m - 1} \right)\phi \left( {x - d_{GI}^{\prime} } \right)\phi \left( y \right)\left[ {{\Phi }\left( y \right)} \right]^{m - 2}$$

From the above model, one can derive the probabilities of the three eyewitness outcomes in culprit-present lineups. Unlike culprit-absent lineups, one cannot easily derive all probabilities from their conceptual definitions. One may only do so for deriving rejection rates but not for deriving suspect and filler identification rates in culprit-present lineups. Below we derive the probabilities using integral calculus.

$$\Pr \left( {REJ{|}G} \right) = \int \limits_{ - \infty }^{\gamma } \mathop \int \limits_{ - \infty }^{\gamma } f_{{ss_{G} }} \left( x \right)f_{{fs_{max} }} \left( y \right)\text{d}x \space\text{d}y = \int \limits_{ - \infty }^{\gamma } f_{{ss_{G} }} \left( x \right){\text{d}}x\int \limits_{ - \infty }^{\gamma } f_{{fs_{max} }} \left( y \right){\text{d}}y{ } = \int \limits_{ - \infty }^{\gamma } \phi \left( {x - d_{GI}^{\prime} } \right){\text{d}}x\mathop \int \limits_{ - \infty }^{\gamma } \left( {m - 1} \right)\left[ {{\Phi }\left( y \right)} \right]^{m - 2} \phi \left( y \right){\text{d}}y = {\Phi }\left( {\gamma - d_{GI}^{\prime} } \right) \cdot {\Phi }\left( \gamma \right)^{m - 1}$$

$$\Pr \left( {IDS|G} \right) = \int\limits_{\gamma }^{ + \infty } {\int\limits_{ - \infty }^{x} {f_{{ss_{G} }} \left( x \right)f_{{fs_{\max } }} \left( y \right)\text{d}y \space\text{d}x = \int\limits_{\gamma }^{ + \infty } {f_{{ss_{G} }} \left( x \right)\int\limits_{ - \infty }^{x} {\text{d}F_{{fs_{\max } }} (y)\space \text{d}x = \int\limits_{\gamma }^{ + \infty } {f_{{ss_{G} }} \left( x \right)\Phi \left( x \right)^{m - 1} \text{d}x = \int\limits_{\gamma }^{ + \infty } {\phi \left( {x - d_{GI}^{\prime} } \right)\Phi \left( x \right)^{m - 1} \text{d}x = \int\limits_{\gamma }^{ + \infty } {\Phi \left( x \right)^{m - 1} \text{d}\Phi \left( {x - d_{GI}^{\prime} } \right)} } } } } } }$$

$$\Pr \left( {IDF|G} \right) = \mathop \int \limits_{\gamma }^{ + \infty } \mathop \int \limits_{ - \infty }^{y } f_{{ss_{G} }} \left( x \right)f_{{fs_{max} }} \left( y \right){\text{d}}x \space {\text{d}}y = \mathop \int \limits_{\gamma }^{ + \infty } f_{{fs_{max} }} \left( y \right)\mathop \int \limits_{ - \infty }^{y } \phi \left( {x - d_{GI}^{\prime} } \right){\text{d}}x \space {\text{d}}y = \mathop \int \limits_{\gamma }^{ + \infty } f_{{fs_{max} }} \left( y \right){\Phi }\left( {y - d_{GI}^{\prime} } \right){\text{d}}y = \mathop \int \limits_{\gamma }^{ + \infty } {\Phi }\left( {y - d_{GI}^{\prime} } \right){\text{d}} {{\Phi }\left( y \right)} ^{m - 1}$$

From the above equations, the ratio between guilty suspect identification and filler identification rates is not equal to $\frac{1}{m-1}$, which is the ratio between innocent suspect identification and filler identification rates in culprit-absent lineups. It indicates that the guilty suspect does not have an equal chance of being identified as a filler. Yet, the mathematical constraint that all probabilities sum up to 1 still holds.

$$\Pr \left( {IDS|G} \right) + \Pr \left( {IDF|G} \right) = \mathop \int \limits_{\gamma }^{ + \infty } \Phi \left( x \right)^{m - 1} \text{d}\Phi \left( {x - d_{GI}^{\prime} } \right) + \mathop \int \limits_{\gamma }^{ + \infty } \Phi \left( {x - d_{GI}^{\prime} } \right)\text{d}\Phi \left( x \right)^{m - 1} = \left[ {\Phi \left( {x - d_{GI}^{\prime} } \right) \Phi \left( x \right)^{m - 1} } \right]_{\gamma }^{ + \infty } = 1 - \Phi \left( {\gamma - d_{GI}^{\prime} } \right) \Phi \left( \gamma \right)^{m - 1} = 1 - \Pr (REJ|G)$$

## Appendix C: Derive ***γ*** and ${{\varvec{d}}}_{{\varvec{G}}{\varvec{I}}}^{\boldsymbol{^{\prime}}}$ from Probabilities of Eyewitness Outcomes

From the above derivations of probabilities of eyewitness outcomes, one can derive witnesses’ decision criterion *γ* from the rejection rate in culprit-absent lineups.

$$\text{Pr}\left(REJ|I\right)={\Phi \left(\gamma \right)}^{m}\boldsymbol{ }\Rightarrow \gamma ={\Phi }^{-1}\left[\sqrt[m]{\text{Pr}\left(REJ|I\right)}\right]={\Phi }^{-1}\left[{\text{Pr}(REJ|I)}^\frac{1}{m}\right]$$

One can then derive the discriminability ${d}^{\prime}_{GI}$ from rejection rates in both culprit-absent and culprit-present lineups.

$$\text{Pr}\left(REJ|G\right)=\Phi \left(\gamma -{d}_{GI}^{\prime}\right) {\Phi \left(\gamma \right)}^{m-1}$$

$$\Rightarrow\Phi \left(\gamma -{d}_{GI}^{\prime}\right)=\frac{\text{Pr}\left(REJ|G\right)}{{\Phi \left(\gamma \right)}^{m-1}}=\frac{\text{Pr}\left(REJ|G\right)}{{\text{Pr}\left(REJ|I\right)}^{\frac{m-1}{m}}}$$

$$\Rightarrow {d}_{GI}^{\prime}=\gamma -{\Phi }^{-1}\left[\frac{\text{Pr}\left(REJ|G\right)}{{\text{Pr}\left(REJ|I\right)}^{\frac{m-1}{m}}}\right]={\Phi }^{-1}\left[{\text{Pr}\left(REJ|I\right)}^\frac{1}{m}\right]-{\Phi }^{-1}\left[{\text{Pr}\left(REJ|I\right)}^\frac{1}{m}\frac{\text{Pr}\left(REJ|G\right)}{\text{Pr}\left(REJ|I\right)}\right]$$

Therefore, one can estimate witnesses’ decision criterion *γ* and discriminability ${d}_{GI}^{\prime}$ from the probabilities of eyewitness outcomes using the two equations below.

## Equation 1.1. Decision criterion*** γ***

$$\gamma ={\Phi }^{-1}\left[\sqrt[m]{\text{Pr}\left(REJ|I\right)}\right]={\Phi }^{-1}\left[{\text{Pr}(REJ|I)}^\frac{1}{m}\right]$$

## Equation 1.2. Discriminability ${{\varvec{d}}}_{{\varvec{G}}{\varvec{I}}}^{\boldsymbol{^{\prime}}}$

$${d}_{GI}^{\prime}={\Phi }^{-1}\left[{\text{Pr}\left(REJ|I\right)}^\frac{1}{m}\right]-{\Phi }^{-1}\left[{\text{Pr}\left(REJ|I\right)}^\frac{1}{m}\frac{\text{Pr}\left(REJ|G\right)}{\text{Pr}\left(REJ|I\right)}\right]$$

Again, *m* denotes the line size, and $(m-1)$ denotes the filler size. $\Phi (*)$ denotes the cdf of a standard normal distribution, and ${\Phi }^{-1}(*)$ denotes its inverse function.

## Appendix D: Assume Unequal Variances and Criterion Shift

We modify some of the assumptions used in the original model to accommodate more sophisticated considerations. For the signal distributions, one may assume that the variance of guilty suspect distribution may not be the same as those of the innocent suspect and filler distributions.

$${ss}_{i}|G \overset{\mathrm{i.i.d.}}{\sim} \mathcal N({d}_{GI}^{\prime}, {\sigma }_{G}^{2})$$

$${ss}_{i}|I \overset{\mathrm{i.i.d.}}{\sim} \mathcal N(0, 1)$$

$${fs}_{ij} \overset{\mathrm{i.i.d.}}{\sim} \mathcal N(0, 1)$$

One can also assume that witnesses may shift their decision criterion across culprit-present and culprit-absent lineups. Specifically, if witnesses use the decision criterion γ in culprit-absent lineups, we assume they would use the decision criterion $(\gamma +\Delta \gamma )$ in culprit-present lineups. $\Delta \gamma$ denotes the amount of criterion shift from culprit-absent lineups to culprit-present lineups. Note that the original model, which assumes equal variance and no criterion shift, is a special case of this more generic model when ${\sigma }_{G}^{2}=1$ and $\Delta \gamma =0$.

Because the joint distribution of innocent suspect and filler signals is not influenced by these modified assumptions, the probabilities of eyewitness outcomes in culprit-absent lineups stay the same as those in the original equal-variance consistent-criterion model.

$$\text{Pr}\left(REJ|I\right)={\Phi \left(\gamma \right)}^{m}$$

$$\text{Pr}\left(IDS|I\right)=\frac{1}{m}\left[1-\Phi {\left(\gamma \right)}^{m}\right]$$

$$\text{Pr}\left(IDF|I\right)=\frac{m-1}{m}\left[1-\Phi {\left(\gamma \right)}^{m}\right]$$

The joint distribution of guilty suspect and filler signals, however, is influenced by these modified assumptions. As a result, the probabilities of eyewitness outcomes in culprit-absent lineups change. But the derivations stay pretty much the same.

$$\Pr \left( {REJ{|}G} \right) = \mathop \int \limits_{ - \infty }^{{\gamma + {\Delta }\gamma }} \mathop \int \limits_{ - \infty }^{{\gamma + {\Delta }\gamma }} f_{{ss_{G} }} \left( x \right)f_{{fs_{max} }} \left( y \right){\text{d}}x{\text{ d}}y = \mathop \int \limits_{ - \infty }^{{\gamma + {\Delta }\gamma }} f_{{ss_{G} }} \left( x \right){\text{d}}x\mathop \int \limits_{ - \infty }^{{\gamma + {\Delta }\gamma }} f_{{fs_{max} }} \left( y \right){\text{d}}y{ } = \mathop \int \limits_{ - \infty }^{{\gamma + {\Delta }\gamma }} \frac{1}{{\sigma_{G} }}\phi \left( {\frac{{x - d_{GI}^{\prime} }}{{\sigma_{G} }}} \right){\text{d}}x\mathop \int \limits_{ - \infty }^{{\gamma + {\Delta }\gamma }} \left( {m - 1} \right)\left[ {{\Phi }\left( y \right)} \right]^{m - 2} \phi \left( y \right){\text{d}}y = {\Phi }\left( {\frac{{\gamma + {\Delta }\gamma - d_{GI}^{\prime} }}{{\sigma_{G} }}} \right) {\Phi }\left( {\gamma + {\Delta }\gamma } \right)^{m - 1}$$

$$\Pr \left( {IDS|G} \right) = \int\limits_{\gamma + \Delta \gamma }^{ + \infty } {\int\limits_{ - \infty }^{x} {_{{ss_{G} }} \left( x \right)f_{{fs_{\max } }} \left( y \right)\text{d}y \text{ d}x = \int\limits_{\gamma + \Delta \gamma }^{ + \infty } {_{{ss_{G} }} \left( x \right)\int\limits_{ - \infty }^{x} {\text{d}F_{{fs_{\max } }} \left(y\right) \space \text{d}x = \int\limits_{\gamma + \Delta \gamma }^{ + \infty } {f_{{ss_{G} }} \left( x \right)\Phi \left( x \right)^{m - 1} \text{d}x = \int\limits_{\gamma + \Delta \gamma }^{ + \infty } {\frac{1}{{\sigma_{G} }}\phi \left( {\frac{{x - d_{GI}^{\prime} }}{{\sigma_{G} }}} \right)\Phi \left( x \right)^{m - 1} \text{d}x = \int\limits_{\gamma + \Delta \gamma }^{ + \infty } {\Phi \left( x \right)^{m - 1} \text{d}\Phi \left( {\frac{{x - d_{GI}^{\prime} }}{{\sigma_{G} }}} \right)} } } } } } }$$

$$\Pr \left( {IDF{|}G} \right) = \mathop \int \limits_{{\gamma + {\Delta }\gamma }}^{ + \infty } \mathop \int \limits_{ - \infty }^{y} f_{{ss_{G} }} \left( x \right)f_{{fs_{max} }} \left( y \right){\text{d}}x \space {\text{d}}y = \mathop \int \limits_{{\gamma + {\Delta }\gamma }}^{ + \infty } f_{{fs_{max} }} \left( y \right)\mathop \int \limits_{ - \infty }^{y } \frac{1}{{\sigma_{G} }}\phi \left( {\frac{{x - d_{GI}^{\prime} }}{{\sigma_{G} }}} \right){\text{d}}x \space {\text{d}}y = \mathop \int \limits_{{\gamma + {\Delta }\gamma }}^{ + \infty } f_{{fs_{max} }} \left( y \right){\Phi }\left( {\frac{{y - d_{GI}^{\prime} }}{{\sigma_{G} }}} \right){\text{d}}y = \mathop \int \limits_{{\gamma + {\Delta }\gamma }}^{ + \infty } {\Phi }\left( {\frac{{y - d_{GI}^{\prime} }}{{\sigma_{G} }}} \right){\text{d}} {{\Phi }\left( y \right)} ^{m - 1}$$

## Derive γ and ${\mathbf{d}}_{\mathbf{G}\mathbf{I}}^{\mathbf{^{\prime}}}$ from Probabilities of Eyewitness Outcomes

From the above derivations of probabilities of eyewitness outcomes, we can derive witnesses’ decision criterion *γ* in culprit-absent lineups, which is the same as the one in the original equal-variance consistent-criterion model.

$$\text{Pr}\left(REJ|I\right)={\Phi \left(\gamma \right)}^{m} \Rightarrow \gamma ={\Phi }^{-1}\left[\sqrt[m]{\text{Pr}\left(REJ|I\right)}\right]={\Phi }^{-1}\left[{\text{Pr}(REJ|I)}^\frac{1}{m}\right]$$

We can then derive discriminability ${d}_{GI}^{\prime}$ when assuming an unequal variance ${\sigma }_{G}^{2}$ for the guilty suspect distribution and a criterion shift $\Delta \gamma$ between the culprit-absent and culprit-present lineups.

$$\text{Pr}\left(REJ|G\right)=\Phi \left(\frac{\gamma +\Delta \gamma -{d}_{GI}^{\prime}}{{\sigma }_{G}}\right) {\Phi \left(\gamma +\Delta \gamma \right)}^{m-1}$$

$$\Rightarrow\Phi \left(\frac{\gamma +\Delta \gamma -{d}_{GI}^{\prime}}{{\sigma }_{G}}\right)=\frac{\text{Pr}\left(REJ|G\right)}{{\Phi \left(\gamma +\Delta \gamma \right)}^{m-1}}$$

$$\Rightarrow {d}_{GI}^{\prime}=(\gamma +\Delta \gamma )-{\sigma }_{G}{\Phi }^{-1}\left[\frac{\text{Pr}\left(REJ|G\right)}{{\Phi \left(\gamma +\Delta \gamma \right)}^{m-1}}\right]$$

Therefore, one can estimate witnesses’ decision criterion *γ* and (unstandardized) discriminability ${d}_{GI}^{\prime}$ from the probabilities of eyewitness outcomes using the two equations below.

## Equation 2.1. Decision criterion*** γ*** in culprit-absent lineups in the unequal-variance criterion-shift model

$$\gamma ={\Phi }^{-1}\left[\sqrt[m]{\text{Pr}\left(REJ|I\right)}\right]={\Phi }^{-1}\left[{\text{Pr}(REJ|I)}^\frac{1}{m}\right]$$

## Equation 2.2. Discriminability ${{\varvec{d}}}_{{\varvec{G}}{\varvec{I}}}^{\boldsymbol{^{\prime}}}$ in the unequal-variance criterion-shift model

$${d}_{GI}^{\prime}=\left(\gamma +\Delta \gamma \right)-{\sigma }_{G}{\Phi }^{-1}\left[\frac{\text{Pr}(REJ|G)}{\Phi {\left(\gamma +\Delta \gamma \right)}^{m-1}}\right]$$

Again, *m* denotes the line size, and $(m-1)$ denotes the filler size. $\Phi (*)$ denotes the cdf of a standard normal distribution, and ${\Phi }^{-1}(*)$ denotes its inverse function.

Figure A1 visualizes the joint distributions under these modified assumptions. Note that the criterion shift component is similar to the Ensemble model depicted in Fig. 11 in the manuscript.

## Appendix E: Assume Correlated Memory Signals

The original model assumes independence among memory signals. This assumption, though, may not hold because lineup members often share some common features (Wixted et al., 2018). Particularly, the correlations among lineup member signals would increase as they become more similar to each other. In other words, whereas one witness may have comparably strong signals for all lineup members, another witness may have weak signals for all lineup members.

In terms of model assumptions, lineup member signals would not follow independent normal distributions any further. Instead, we could assume that their signals follow a multivariate normal distribution, which allows for nonzero correlations among memory signals from the same witness. For culprit-absent lineups,

$${\left[\begin{array}{c}ss\\ \begin{array}{c}f{s}_{1}\\ f{s}_{2}\\ \begin{array}{c}\vdots \\ f{s}_{j}\end{array}\end{array}\end{array}\right]}_{i} \overset{\mathrm{i.i.d.}}{\sim} \; \mathcal{MVN}\left(\varvec{{\mu }_{CA}}=\left[\begin{array}{c}0\\ 0\\ \begin{array}{c}0\\ \vdots \\ 0\end{array}\end{array}\right], \; \Sigma =\left[\begin{array}{ccc}1& \rho & \begin{array}{ccc}\rho & \dots & \rho \end{array}\\ \rho & 1& \begin{array}{ccc}\rho & \dots & \rho \end{array}\\ \begin{array}{c}\rho \\ \vdots \\ \rho \end{array}& \begin{array}{c}\rho \\ \vdots \\ \rho \end{array}& \begin{array}{c}\begin{array}{ccc}1& \dots & \rho \end{array}\\ \begin{array}{ccc}\vdots & \ddots & \vdots \end{array}\\ \begin{array}{ccc}\rho & \dots & 1\end{array}\end{array}\end{array}\right]\right)$$

For culprit-present lineups,

$${\left[\begin{array}{c}ss\\ \begin{array}{c}f{s}_{1}\\ f{s}_{2}\\ \begin{array}{c}\vdots \\ f{s}_{j}\end{array}\end{array}\end{array}\right]}_{i} \overset{\mathrm{i.i.d.}}{\sim} \; \mathcal{MVN}\left(\varvec{\mu _{CP}}=\left[\begin{array}{c}{d}_{GI}^{\prime}\\ 0\\ \begin{array}{c}0\\ \vdots \\ 0\end{array}\end{array}\right], \; \Sigma =\left[\begin{array}{ccc}{\sigma }_{G}^{2}& \rho {\sigma }_{G}& \begin{array}{ccc}\rho {\sigma }_{G}& \dots & \rho {\sigma }_{G}\end{array}\\ \rho {\sigma }_{G}& 1& \begin{array}{ccc}\rho & \dots & \rho \end{array}\\ \begin{array}{c}\rho {\sigma }_{G}\\ \vdots \\ \rho {\sigma }_{G}\end{array}& \begin{array}{c}\rho \\ \vdots \\ \rho \end{array}& \begin{array}{c}\begin{array}{ccc}1& \dots & \rho \end{array}\\ \begin{array}{ccc}\vdots & \ddots & \vdots \end{array}\\ \begin{array}{ccc}\rho & \dots & 1\end{array}\end{array}\end{array}\right]\right)$$

In the above assumptions, *ρ* denotes the correlations among lineup member signals. ${d}_{GI}^{\prime}$ denotes the unstandardized difference between the marginal means of guilty suspect and innocent suspect distributions. ${\sigma }_{G}^{2}$ denotes the variance of the guilty suspect distribution.

Now let us derive the pdf of the joint distribution of suspect and max filler signals. We derive the pdf for the more generic situation of culprit-present lineups, as its distribution allows for flexible mean and variance.

For culprit-present lineups,

$${\left[\begin{array}{c}ss\\ \begin{array}{c}f{s}_{1}\\ f{s}_{2}\\ \begin{array}{c}\vdots \\ f{s}_{j}\end{array}\end{array}\end{array}\right]}_{i} \overset{\mathrm{i.i.d.}}{\sim} \; \mathcal{MNV} \left({{\varvec{\mu}}}_{{\varvec{C}}{\varvec{P}}}=\left[\begin{array}{c}{d}_{GI}^{\prime}\\ 0\\ \begin{array}{c}0\\ \vdots \\ 0\end{array}\end{array}\right], \; \Sigma =\left[\begin{array}{ccc}{\sigma }_{G}^{2}& \rho {\sigma }_{G}& \begin{array}{ccc}\rho {\sigma }_{G}& \dots & \rho {\sigma }_{G}\end{array}\\ \rho {\sigma }_{G}& 1& \begin{array}{ccc}\rho & \dots & \rho \end{array}\\ \begin{array}{c}\rho {\sigma }_{G}\\ \vdots \\ \rho {\sigma }_{G}\end{array}& \begin{array}{c}\rho \\ \vdots \\ \rho \end{array}& \begin{array}{c}\begin{array}{ccc}1& \dots & \rho \end{array}\\ \begin{array}{ccc}\vdots & \ddots & \vdots \end{array}\\ \begin{array}{ccc}\rho & \dots & 1\end{array}\end{array}\end{array}\right]\right)$$

Or equivalently,

$${\left[\begin{array}{c}{\varvec{ss}}\\ \varvec{fs}\end{array}\right]}_{i} \overset{\mathrm{i.i.d.}}{\sim}\; \mathcal {MNV}\left(\varvec{\mu_{CP}} = \left[\begin{array}{c}\varvec{\mu_{ss}} =d_{GI}^{\prime}\\ \varvec{\mu_{fs}} =\varvec{0_{m-1}}\end{array}\right],\; \Sigma =\left[\begin{array}{cc}{\Sigma_{\varvec{ss}}} & {\Sigma_{\varvec{ss,fs}}}\\ {\Sigma_{\varvec{fs,ss}}} & {\Sigma_{\varvec{fs}}}\end{array}\right]\right)$$

In the above assumption, *m* denotes the lineup size, and (*m*−1) denotes the filler size. The variance and covariance matrices are,

$$\begin{array}{cc}{\Sigma }_{\varvec{ss}}={\sigma }_{G}^{2}& {\Sigma }_{\varvec{ss,fs}}=\rho {\sigma }_{G}\varvec{1^T_{m-1}} \\ {\Sigma }_{\varvec{fs,ss}}=\rho {\sigma_G}\varvec{1_{m-1}} & {\Sigma }_{\varvec{fs}} = (1-\rho ){{\varvec{I_{m-1}}}} + \rho \varvec{1_{m-1}} \varvec{1^T_{m-1}}\end{array}$$

## Conditional Distribution of Filler Signals

We first derive the pdf for the conditional distribution of all filler signals given the suspect signal. From the property of a multivariate normal distribution, the conditional distribution is also a multivariate normal distribution (Casella & Berger, 2002).

$${\varvec{f}}{\varvec{s}}|{\varvec{s}}{\varvec{s}} \sim \mathcal{MVN}\left({{\varvec{\mu}}}_{{\varvec{f}}{\varvec{s}}|{\varvec{s}}{\varvec{s}}},\; {\Sigma }_{{\varvec{f}}{\varvec{s}}|{\varvec{s}}{\varvec{s}}}\right)$$

$${{\varvec{\mu}}}_{{\varvec{f}}{\varvec{s}}|{\varvec{s}}{\varvec{s}}} = \varvec{\mu_{fs}} + {\Sigma }_{\varvec{fs,ss}} \Sigma_{\varvec{ss}}^{-1} ({\varvec{ss}}-{\varvec{\mu_{ss}}})= \varvec{0_{m-1}} +\rho {\sigma }_{G} \varvec{1_{m-1}} \frac{1}{\sigma_G^2}(ss-d_{GI}^{\prime})=\rho \frac{(ss-{d}_{GI}^{\prime})}{{\sigma }_{G}}\varvec{1_{m-1}}$$

$$\Sigma_{\varvec{fs|ss}} = \Sigma_{\varvec{fs}} - \Sigma_{\varvec{fs,ss}} \Sigma{\varvec{ss}}^{-1}{\Sigma_{\varvec{ss,fs}}} = \left[\left(1-\rho \right)\varvec{I_{m-1}} + \rho \varvec{1_{m-1}}\varvec{1_{m-1}^T} \right] - \rho {\sigma_G}{\varvec{1_{m-1}}} \frac{1}{\sigma_G^2} \rho {\sigma_G}{\varvec{1_{m-1}^T}} = \left[\begin{array}{ccc}1& \rho & \begin{array}{cc}\dots & \rho \end{array}\\ \rho & 1& \begin{array}{cc}\dots & \rho \end{array}\\ \begin{array}{c}\vdots \\ \rho \end{array}& \begin{array}{c}\vdots \\ \rho \end{array}& \begin{array}{cc}\ddots & \vdots \\ \rho & 1\end{array}\end{array}\right]-\left[\begin{array}{ccc}{\rho }^{2}& {\rho }^{2}& \begin{array}{cc}\dots & {\rho }^{2}\end{array}\\ {\rho }^{2}& {\rho }^{2}& \begin{array}{cc}\dots & {\rho }^{2}\end{array}\\ \begin{array}{c}\vdots \\ {\rho }^{2}\end{array}& \begin{array}{c}\vdots \\ {\rho }^{2}\end{array}& \begin{array}{cc}\ddots & \vdots \\ {\rho }^{2}& {\rho }^{2}\end{array}\end{array}\right]=\left[\begin{array}{ccc}1-{\rho }^{2}& \rho -{\rho }^{2}& \begin{array}{cc} \dots & \rho -{\rho }^{2}\end{array}\\ \rho -{\rho }^{2}& 1-{\rho }^{2}& \begin{array}{cc} \dots & \rho -{\rho }^{2}\end{array}\\ \begin{array}{c}\vdots \\ \rho -{\rho }^{2}\end{array}& \begin{array}{c}\vdots \\ \rho -{\rho }^{2}\end{array}& \begin{array}{cc} \ddots & \vdots \\ \rho -{\rho }^{2}& 1-{\rho }^{2}\end{array}\end{array}\right]= \; \left(1-\rho \right){\varvec{I_{m-1}}}+(\rho -{\rho }^{2}){\varvec{1_{m-1}}}{\varvec{1_{m-1}^T}}$$

Because this conditional distribution is a multivariate normal distribution, the marginal conditional distribution of each filler would be a normal distribution.

$$f{s}_{j}|ss \sim \mathcal{N} \left(\rho \frac{(ss-{d}_{GI}^{\prime})}{{\sigma }_{G}}, \; 1-{\rho }^{2}\right)$$

## Conditional Distribution of Max Filler Signals

We then derive the pdf for the conditional distribution of the max filler signals given the suspect signal. According to Corollary 5 in Arellano-Valle and Genton (2008), one can derive the pdf of the max filler signals as all filler signals are an exchangeable random vector with a multivariate normal distribution.

$${f}_{f{s}_{max}|ss}\left(x|y\right)=\left(m-1\right) \times {\phi }_{1}\left(x;\; \mu =\rho \frac{\left(y-{d}_{GI}^{\prime}\right)}{{\sigma }_{G}},\; {\sigma }^{2}=1-{\rho }^{2}\right)\times {\varvec{\Phi_{m-2}}} \left(\sqrt{1-(\rho -{\rho }^{2})}z\varvec{1_{m-2}} ;\; {\varvec{\mu}}=\varvec{0_{m-2}}, \; \Sigma = \varvec{I_{m-2}} + (\rho -{\rho }^{2}) \varvec{1_{m-2}} \varvec{1_{m-2}^T} \right)$$

where $z=\frac{x-\mu }{\sigma }=\frac{x-\rho \frac{\left(y-{d}_{GI}^{\prime}\right)}{\sigma_G}}{\sqrt{1-{\rho }^2}}$, ${\phi }_{1}$ is the pdf of the marginal conditional distribution of one filler conditioning on suspect signals, and $\varvec{\Phi_{m-2}}$ is the cdf of the joint distribution of $(m-2)$ fillers.

## Joint Distribution of Suspect and Max Filler Signals

With knowledge of the conditional distribution of max filler signals and the marginal distribution of suspect signals, one can obtain their joint distribution.

$${f}_{f{s}_{max},ss}\left(x,y\right)={f}_{f{s}_{max}|ss}\left(x|y\right){f}_{ss}\left(y\right)=\left(m-1\right) \times {\phi }_{1}\left(x;\; \rho \frac{\left(y-{d}_{GI}^{\prime}\right)}{\sigma_G},\; 1-{\rho }^{2}\right) \times \varvec{\Phi_{m-2}} \left(\sqrt{1-\left(\rho -{\rho }^{2}\right)}z \varvec{1_{m-2}};\;{\varvec{\mu}} = \varvec{0_{m-2}} ,\; \Sigma =\varvec{I_{m-2}} + \left(\rho - {\rho }^{2}\right)\varvec{1_{m-2}} \varvec{1_{m-2}^T}\right) \times {\phi_2}(y;\;{d}_{GI}^{\prime}, {\sigma }_{G}^{2})$$

where $z=\frac{x-\mu}{\sigma} = \frac{x-\rho \frac{\left(y-{d}_{GI}^{\prime}\right)}{\sigma_G}} {\sqrt{1-{\rho }^2}}$, ${\phi }_{1}$ is the pdf of the marginal conditional distribution of one filler conditioning on suspect signals, $\varvec{\Phi_{m-2}}$ is the cdf of the joint distribution of $(m-2)$ fillers, and ${\phi }_{2}$ is the pdf of the marginal distribution of suspect signals.

When the suspect signals come from an innocent suspect (i.e., culprit-absent lineups), the joint pdf is a special case of the above joint pdf with ${d}_{GI}^{\prime}=0$ and ${\sigma }_{G}^{2}=1$. In other words, the joint pdf will reduce to,

$${f}_{{fs}_{max},ss}\left(x,y\right)={f}_{{fs}_{max}|ss}\left(x|y\right){f}_{ss}\left(y\right)=\left(m-1\right) \times {\phi }_{1}\left(x;\; \rho y,\; 1-{\rho }^{2}\right) \times \varvec{\Phi_{m-2}} \left(\sqrt{1-\left(\rho -{\rho }^{2}\right)}z \varvec{1_{m-2}};\; {\varvec{\mu}}=\varvec{0_{m-2}},\; \Sigma =\varvec{I_{m-2}} + \left(\rho - {\rho }^{2}\right)\varvec{1_{m-2}} \varvec{1_{m-2}^T}\right)\times {\phi }_{2}(y;\; 0, 1)$$

where $z=\frac{x-\mu }{\sigma }=\frac{x-\rho y}{\sqrt{1-{\rho }^{2}}}$, ${\phi }_{1}$ is the pdf of the marginal conditional distribution of one filler conditioning on innocent suspect signals, $\varvec{\Phi_{m-2}}$ is the cdf of the joint distribution of $(m-2)$ fillers, and ${\phi }_{2}$ is the pdf of the marginal distribution of innocent suspect signals.

## Appendix F: Ensemble Model Approximation

The Ensemble model treats the difference between a lineup member’s signal and the average signal of all lineup members as the decision variable and applies the MAX decision rule to the difference scores (Wixted et al., 2018). In fact, these assumptions can be approximated as applying the MAX decision rule to the original memory signals with a criterion shift between culprit-present and culprit-absent lineups.

Applying the MAX decision rule to the difference between the suspect’s memory signal and the average memory signal, one would have,

$$ss-\frac{1}{m}\left(ss+f{s}_{1}+\dots +f{s}_{m-1}\right)>\gamma$$

$$\Rightarrow ss>\gamma +\frac{1}{m}\left(ss+f{s}_{1}+\dots +f{s}_{m-1}\right)=\gamma +\frac{1}{m}\left[\left({\mu }_{ss}+{\varepsilon }_{ss}\right)+\left({\mu }_{F}+{\varepsilon }_{f{s}_{1}}\right)+\dots \left({\mu }_{F}+{\varepsilon }_{f{s}_{m-1}}\right)\right]=\gamma +\frac{1}{m}\left[\left({\mu }_{ss}+{\varepsilon }_{ss}\right)+\left(0+{\varepsilon }_{f{s}_{1}}\right)+\dots \left(0+{\varepsilon }_{f{s}_{m-1}}\right)\right]=\gamma +\frac{1}{m}\left[\left({\mu }_{ss}+{\varepsilon }_{ss}\right)+{\varepsilon }_{f{s}_{1}}+\dots {\varepsilon }_{f{s}_{m-1}}\right]\approx \gamma +\frac{1}{m}\left[\left({\mu }_{ss}+0\right)+0+\dots 0\right]=\gamma +\frac{{\mu }_{ss}}{m}$$

Similarly, applying the MAX decision rule to the difference between the max filler’s memory signal and the average memory signal, one would have,

$${fs}_{max}-\frac{1}{m}\left(ss+f{s}_{1}+\dots +f{s}_{m-1}\right)>\gamma$$

$$\Rightarrow {fs}_{max}>\gamma +\frac{1}{m}\left(ss+f{s}_{1}+\dots +f{s}_{m-1}\right)\approx \gamma +\frac{{\mu }_{ss}}{m}$$

In the above derivation, *m* denotes the lineup size. ${\mu }_{ss}$ denotes the population mean of suspect signals. Specifically, ${\mu }_{ss}={\mu }_{I}$ for culprit-absent lineups, and ${\mu }_{ss}={\mu }_{G}$ for culprit-present lineups. ${\mu }_{F}$ denotes the population mean of filler signals, and ${\mu }_{F}=0$ according to the model assumptions. $\varepsilon$'s denote the random errors of memory signals. According to the model assumptions, $\varepsilon \sim \mathcal N(0, {\sigma }^{2})$. Therefore, the random errors are approximated as zeros in the above derivation.

When witnesses view culprit-absent lineups, that is, when the suspect signal comes from an innocent suspect, the above conditions can be transformed to,

$$ss|I>\gamma +\frac{{\mu }_{I}}{m}$$

$${fs}_{max}>\gamma +\frac{{\mu }_{I}}{m}$$

When witnesses view culprit-present lineups, that is, when the suspect signal comes from a guilty suspect, the above conditions can be transformed to,

$$ss|G>\gamma +\frac{{\mu }_{G}}{m}$$

$${fs}_{max}>\gamma +\frac{{\mu }_{G}}{m}$$

Because ${\mu }_{G}>{\mu }_{I}$, the above decision rule is equivalent to applying the MAX decision rule to the original memory signals, but with different decision criteria for culprit-present and culprit-absent lineups. Particularly, the above decision rule implies a more conservative decision criterion for culprit-present lineups than culprit-absent lineups.
