# Supplementary material for: A multi-item signal detection theory model for eyewitness identification
Source: Cogn Res Princ Implic. 2025 Aug 22;10:54. doi: 10.1186/s41235-025-00652-3 (PMC12373971; doi:10.1186/s41235-025-00652-3)
Supplement: Supplementary file 1 — Additional file 1. [file 41235_2025_652_MOESM1_ESM.docx]

**A Multi-Item Signal Detection Model for Eyewitness Identification**

**Supplementary Materials**

**Data-Model Discrepancy in Applying Univariate SDT to Lineup Decisions**

The lack of a rigorously defined mathematical model could be problematic for the application of SDT to eyewitness research. Specifically, the omission of a mathematical model could lead to discrepancies between models’ verbal descriptions and visual presentations. The visual presentation of a model reflects its underlying mathematical structure and should therefore be derived from a mathematical model (Friendly et al., 2008; Palais, 1999).

As shown in Figure S1, previous eyewitness SDT models are generally built upon univariate SDT (uSDT): They assume univariate distributions for lineup signals and project *separate* filler distributions onto the same univariate decision space used in the binary recognition task, which includes a guilty suspect distribution (i.e., the old-item distribution) and an innocent distribution (i.e., the new-item distribution). Even though uSDT is highly useful for understanding binary recognition tasks, it leads to an imprecise presentation of lineup member distributions and thus may cause confusion when modeling lineup tasks that involve multiple recognition items—one suspect and several fillers.

First, simply projecting a filler distribution on top of the guilty and innocent distributions may give the wrong impression that a lineup task involves *three* signal distributions—one for guilty suspects, one for innocent suspects, and one for fillers. Yet, the nature of lineup tasks determines that only *two* distributions are involved—one for culprit-present lineups and one for culprit-absent lineups, corresponding to the two possible states of ground truth (for more detail see the mSDT model section in the main manuscript). The univariate presentation of three signal distributions thus does not capture the *correct number* of eyewitness signal distributions involved in a lineup task, let alone provide a precise presentation of the distributions.

**Figure S1**

*Application of Univariate SDT to Lineup Tasks*

*
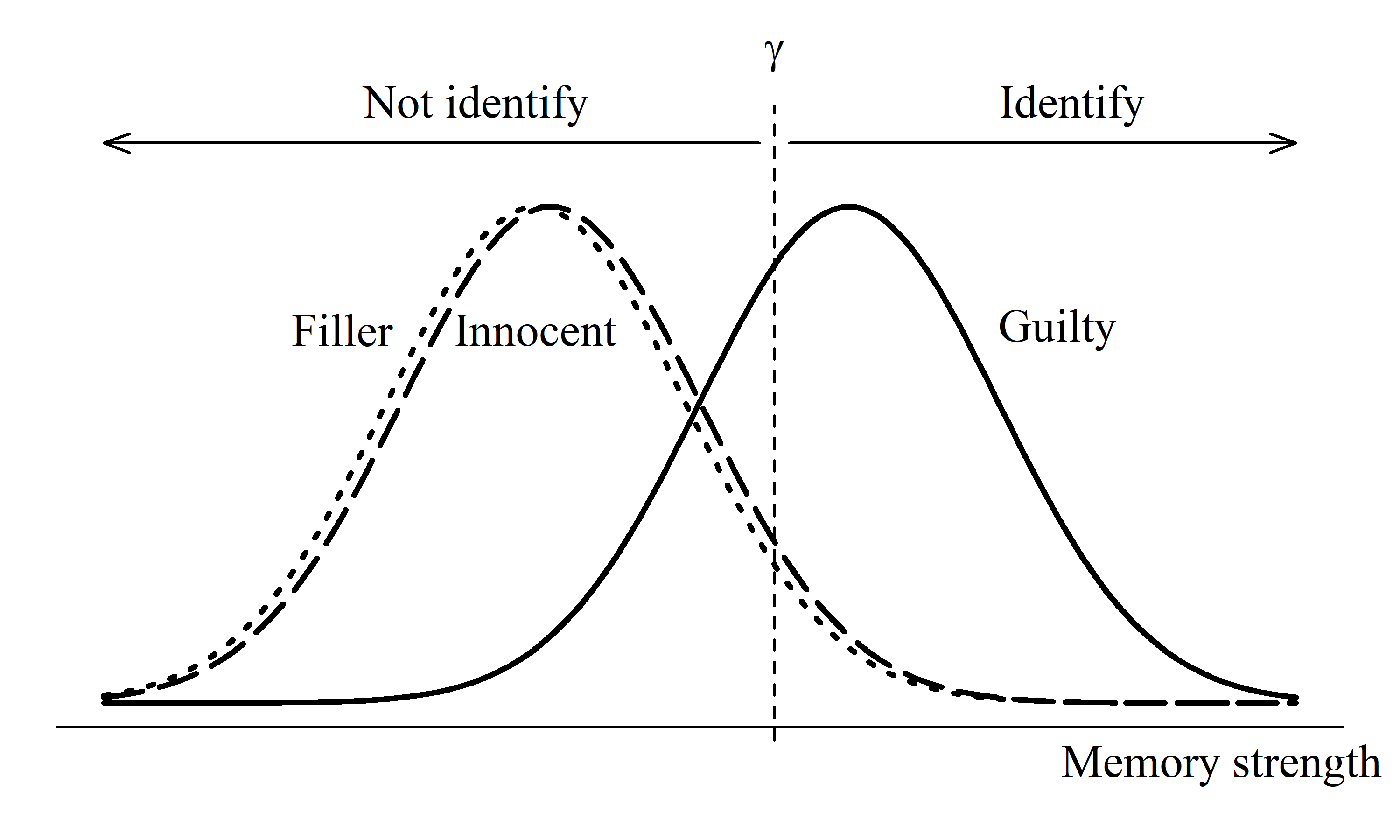
*

*Note.* The solid curve depicts a guilty suspect distribution, the long-dashed curve depicts an innocent suspect distribution, and the dotted-dashed curve depicts a filler distribution. The vertical dashed line depicts the decision criterion $\gamma$.

Related to the above issue, the simple addition of *one* filler distribution also fails to properly present *all* fillers involved in a lineup. It is unclear if the filler distribution presents signals of all fillers or just one filler. If presenting the distribution of all fillers, how could this distribution explain the effects of lineup fillers, such as filler similarity or filler size? If presenting the distribution of one filler, not only is the model limited in explaining the effects of fillers, but the model deviates even further from actual lineup tasks by implying that witnesses would form *seven* signal distributions in a six-member lineup (i.e., five filler distributions plus a guilty suspect distribution and an innocent suspect distribution; or even 12 distributions if one differentiates filler distributions between culprit-present and culprit-absent lineups). In brief, simply projecting a filler distribution onto the univariate space does not properly incorporate fillers and limits the models’ ability to explain how fillers impact eyewitness decisions.

In addition, the decision rules presented in the univariate space deviates from the ones verbally described in previous eyewitness models, leading to predictions inconsistent with actual identification decisions. For example, the decision rule depicted in Figure S1 is the same as the linear decision rule used in uSDT. Such a linear decision rule suggests that a lineup member would be identified if their signal exceeds the decision criterion. According to this visualized decision rule, it is reasonable to infer that witnesses would identify multiple lineup members as long as these lineup members’ signals *all* exceed the criterion. Such a situation does not happen in reality. This is because the decision rules verbally described in previous eyewitness models are more sophisticated and preclude these outrageous predictions. Yet, their visual presentations in a univariate decision space clearly do not match and thus fail to capture the verbal description.

Presenting these signal distributions in the univariate space further confuses how researchers could use SDT to understand and analyze lineup data. In a binary recognition task, the areas under the two distribution curves, separated by a linear decision criterion, correspond to the probabilities of the four decision outcomes. This is because the model provides a precise mathematical account for binary decisions (see Figure 1 in the manuscript).

Such a data-model consistency, however, does not exist in the application of uSDT to lineup data. If the eyewitness SDT models had the same level of data-model consistency as the binary uSDT model, the areas under the univariate distributions should correspond to the response rates of the six eyewitness outcomes (see Table 1 in the main manuscript). Looking at Figure S1, one may think that the area under the guilty suspect distribution beyond the linear decision criterion would correspond to the guilty suspect identification rate, $Pr(IDS|G)$, and the area under the innocent suspect distribution beyond the criterion would correspond to the innocent suspect identification rate, $Pr(IDS|I)$. But which areas correspond to filler identification rates? There is only one filler distribution in Figure S1 but two filler identification rates in reality, $Pr(IDF|G)$ and $Pr(IDF|I)$. The same dilemma occurs with rejection rates. Which areas correspond to rejection rates? If the areas under the guilty and innocent suspect distributions below the decision criterion correspond to the rejection rates, clearly, the model would violate the mathematical constraint that the three response rates sum to 1 under the same ground truth (i.e., $\Pr\left( IDS | G \right)+\Pr\left( IDF | G \right)+\Pr\left( REJ | G \right)=1$ and $\Pr\left( IDS | I \right)+\Pr\left( IDF | I \right)+\Pr\left( REJ | I \right)=1$). These discrepancies, again, suggest that the uSDT models may not provide a precise mathematical account for eyewitness decisions.

In sum, the application of SDT to lineup data has made significant theoretical contributions to understanding and improving eyewitness identification decisions. However, such applications often rely on univariate distributions of lineup recognition items, which could lead to inconsistent predictions with actual lineup decisions. A rigorously defined mathematical model that could properly incorporate all lineup items would greatly benefit the understanding of eyewitness lineup decisions, just like the classic univariate SDT benefits the understanding of binary recognition decisions.

**Estimate Discriminability** $\boldsymbol{d}_{\boldsymbol{GI}}^{\boldsymbol{'}}$ **from Lineup Data**

One beauty of SDT is that it explains a diagnostic system’s decision process with only two simple parameters—discriminability and decision criterion. When applying SDT to recognition memory, researchers typically place a premium on discriminability because it reflects the mean difference between the old and new signals and is not influenced by changes in decision criterion (Banks, 1970).

In the same vein, researchers have advocated using discriminability as a primary measure to quantify eyewitness performance (e.g., Wixted & Mickes, 2012). But how should one estimate discriminability from eyewitness data? For binary tasks such as memory recognition, discriminability can be directly estimated from true and false positive rates (Macmillan & Creelman, 2005). The mSDT model, however, reveals that the same approach does not work for eyewitness lineup data (see Figure 7 in the manuscript). Unlike binary SDT tasks, one cannot accurately estimate discriminability for lineup SDT tasks from guilty and innocent suspect identification rates. This is because fillers draw responses away from suspect identifications (i.e., *filler siphoning*) (Smith et al., 2017; Wells et al., 2015). Such filler siphoning is visualized in Figure 7 in the manuscript with the area in between the dashed red line and the line of equality. The filler siphoning area makes it impossible to estimate discriminability directly from guilty and innocent suspect identification rates. Instead, one needs to estimate discriminability and decision criterion using rejection rates, as shown in Equations 1.1 and 1.2.

We conducted a simulation study to compare the accuracy in estimating the parameters when using equations derived from the mSDT model versus when using equations derived from the classic uSDT model. We first simulated witness responses according to the assumptions that the suspect and filler signals follow independent normal distributions with variances of one and witnesses use the MAX decision rule to make identification decisions. We conducted 10,000 simulations for each combination of different values of discriminability and response criterion under different lineup sizes (see Table S1). Each simulation contained a total of 2,000 witness responses, 1,000 for culprit-present lineups and 1,000 for culprit-absent lineups. For each simulated dataset (*n* = 2,000), we then estimated discriminability and response criterion using the equations derived from mSDT and uSDT, respectively. Thus, a total of 10,000 estimates were generated for each parameter from the simulations. The R script for the simulation is available at osf.io/n2zbc/. The summary statistics of the estimated parameters are displayed in Table S1.

Two main observations emerge from the simulation results. First, the means of the discriminability (*d’*) and response criterion (*γ*) estimated from mSDT are consistently closer to the population parameters compared to the values estimated from uSDT, regardless of lineup size. In other words, the mSDT estimates provide less biased estimates of the population parameters than the uSDT estimates. When such a convergence occurs—the mean of estimated parameters is close to the parameters used for the simulations—it suggests that the model is likely functioning as expected, accurately representing the problem, and producing consistent, reliable results (Law & Kelton, 2000). Therefore, the convergence of the mSDT estimates to the population parameters used for the simulations suggests that the mSDT model accurately represents the lineup identification task.

**Table S1**

*Discriminability and Response Criterion Used for and Estimated from the Simulated Data*

| Lineup size | Estimation method | Estimated parameter |  | Parameters used in simulations | | | | | | | | | | | |
| --- | --- | --- | --- | --- | --- | --- | --- | --- | --- | --- | --- | --- | --- | --- | --- |
|  |  |  |  | $d^{'}=0.5$ | | |  | $d^{'}=1$ | | |  | $d^{'}=1.5$ | | | |
|  |  |  |  | $\gamma=0.5$ | $\gamma=1$ | $\gamma=1.5$ |  | $\gamma=0.5$ | $\gamma=1$ | $\gamma=1.5$ |  | $\gamma=0.5$ | $\gamma=1$ | $\gamma=1.5$ |  |
| 2 | uSDT | $d^{'}$ |  | 0.476 (0.046) | 0.492 (0.052) | 0.498 (0.064) |  | 0.944 (0.047) | 0.983 (0.050) | 0.997 (0.058) |  | 1.403 (0.050) | 1.469 (0.052) | 1.492 (0.058) |  |
|  |  | $\gamma$ |  | 0.641 (0.025) | 1.054 (0.031) | 1.519 (0.042) |  | 0.641 (0.024) | 1.054 (0.031) | 1.519 (0.042) |  | 0.641 (0.024) | 1.054 (0.031) | 1.518 (0.042) |  |
|  | mSDT | $d^{'}$ |  | 0.500 (0.076) | 0.499 (0.076) | 0.500 (0.088) |  | 1.001 (0.071) | 1.001 (0.066) | 1.001 (0.072) |  | 1.502 (0.072) | 1.502 (0.065) | 1.501 (0.067) |  |
|  |  | $\gamma$ |  | 0.500 (0.033) | 1.000 (0.035) | 1.502 (0.044) |  | 0.500 (0.032) | 1.001 (0.035) | 1.502 (0.044) |  | 0.500 (0.032) | 1.000 (0.035) | 1.501 (0.044) |  |
| 4 | uSDT | $d^{'}$ |  | 0.452 (0.043) | 0.479 (0.048) | 0.493 (0.057) |  | 0.895 (0.041) | 0.953 (0.045) | 0.986 (0.051) |  | 1.328 (0.042) | 1.423 (0.044) | 1.476 (0.049) |  |
|  |  | $\gamma$ |  | 0.868 (0.012) | 1.152 (0.019) | 1.552 (0.028) |  | 0.868 (0.012) | 1.152 (0.019) | 1.552 (0.028) |  | 0.868 (0.012) | 1.152 (0.019) | 1.552 (0.028) |  |
|  | mSDT | $d^{'}$ |  | 0.498 (0.124) | 0.497 (0.105) | 0.496 (0.110) |  | 1.001 (0.106) | 0.998 (0.085) | 0.999 (0.081) |  | 1.503 (0.104) | 1.501 (0.075) | 1.500 (0.069) |  |
|  |  | $\gamma$ |  | 0.500 (0.029) | 1.000 (0.027) | 1.501 (0.032) |  | 0.500 (0.029) | 1.000 (0.027) | 1.500 (0.032) |  | 0.500 (0.028) | 1.000 (0.027) | 1.501 (0.032) |  |
| 6 | uSDT | $d^{'}$ |  | 0.443 (0.043) | 0.470 (0.047) | 0.489 (0.054) |  | 0.879 (0.041) | 0.936 (0.042) | 0.978 (0.048) |  | 1.309 (0.041) | 1.394 (0.042) | 1.462 (0.045) |  |
|  |  | $\gamma$ |  | 1.043 (0.007) | 1.240 (0.014) | 1.584 (0.022) |  | 1.043 (0.007) | 1.240 (0.014) | 1.585 (0.022) |  | 1.043 (0.007) | 1.240 (0.013) | 1.584 (0.022) |  |
|  | mSDT | $d^{'}$ |  | 0.490 (0.188) | 0.492 (0.137) | 0.493 (0.134) |  | 0.997 (0.157) | 1.000 (0.103) | 0.998 (0.090) |  | 1.506 (0.153) | 1.501 (0.091) | 1.499 (0.075) |  |
|  |  | $\gamma$ |  | 0.498 (0.030) | 1.000 (0.025) | 1.500 (0.027) |  | 0.499 (0.029) | 1.000 (0.025) | 1.500 (0.027) |  | 0.500 (0.030) | 1.000 (0.024) | 1.500 (0.028) |  |
| 8 | uSDT | $d^{'}$ |  | 0.441 (0.044) | 0.465 (0.047) | 0.487 (0.053) |  | 0.877 (0.041) | 0.923 (0.042) | 0.971 (0.046) |  | 1.309 (0.041) | 1.377 (0.041) | 1.452 (0.044) |  |
|  |  | $\gamma$ |  | 1.183 (0.004) | 1.319 (0.010) | 1.616 (0.018) |  | 1.183 (0.004) | 1.319 (0.010) | 1.616 (0.018) |  | 1.183 (0.004) | 1.319 (0.010) | 1.616 (0.018) |  |
|  | mSDT | $d^{'}$ |  | 0.478 (0.291) | 0.488 (0.176) | 0.490 (0.157) |  | 0.992 (0.232) | 0.996 (0.129) | 0.998 (0.101) |  | 1.513 (0.224) | 1.499 (0.109) | 1.499 (0.081) |  |
|  |  | $\gamma$ |  | 0.498 (0.033) | 1.000 (0.024) | 1.500 (0.025) |  | 0.498 (0.033) | 1.000 (0.024) | 1.500 (0.025) |  | 0.498 (0.033) | 0.999 (0.024) | 1.500 (0.024) |  |

*Note*. The table displays the means and standard deviations (in parentheses) of discriminability and response criterion estimated from the simulated data. A total of 10,000 simulations were conducted for each combination of discriminability and response criterion under each lineup size. Each simulation contained 2,000 witness responses, 1,000 for culprit-present lineups and 1,000 for culprit-absent lineups.

Another observation from the simulation is that the means of discriminability and response criterion estimated from uSDT increasingly deviate from the population parameters as lineup size increases. This again raises concern that uSDT does not adequately fit a lineup task in which fillers are included. Considering that the means of the parameters estimated from mSDT are much closer to the population parameters than those estimated from uSDT, and that the estimated parameter from uSDT increasingly deviate from the population parameters as lineup size increases, it is likely that mSDT better encompasses the eyewitness lineup decision-making task than uSDT.

We conducted further simulations to take potential complexity in data into account. Because considering noises in *d’* is equivalent to a shrank *d’* (i.e., ${d'}_{shrank}=\frac{d^{'}}{\sigma_{noise}}$), we focused on introducing potential noises into response criteria. These simulations followed the procedures described above, but incorporated various levels of criterion variability across witnesses ($\sigma_{\gamma}=0.1$ or $\sigma_{\gamma}=0.5$). The R script for the simulation is available at osf.io/n2zbc/. The summary statistics of the estimated parameters are displayed in Table S2.

From Table S2, mSDT generally provides more accurate parameter estimates than uSDT. However, mSDT estimates are more susceptible to criterion variability, resulting in reduced accuracy as variability increases—a trend exacerbated with larger lineup sizes. By contrast, uSDT estimates remain relatively resistant to the impact of criterion variability.

**Table S2**

*Discriminability and Response Criterion Used for and Estimated from the Simulated Data Considering Criterion Variability*

| Lineup size | Estimation method | Estimated parameter |  | Parameters used in simulations | | | | | | | | |
| --- | --- | --- | --- | --- | --- | --- | --- | --- | --- | --- | --- | --- |
|  |  |  |  | $d^{'}=0.5$ | | | |  | $d^{'}=1$ | | | |
|  |  |  |  | $\gamma=0.5$ | | $\gamma=1$ | |  | $\gamma=0.5$ | | $\gamma=1$ | |
|  |  |  |  | $\sigma_{\gamma}=0.1$ | $\sigma_{\gamma}=0.5$ | $\sigma_{\gamma}=0.1$ | $\sigma_{\gamma}=0.5$ |  | $\sigma_{\gamma}=0.1$ | $\sigma_{\gamma}=0.5$ | $\sigma_{\gamma}=0.1$ | $\sigma_{\gamma}=0.5$ |
| 2 | uSDT | $d^{'}$ |  | 0.474 (0.045) | 0.440 (0.042) | 0.490 (0.051) | 0.448 (0.045) |  | 0.941 (0.046) | 0.879 (0.043) | 0.978 (0.049) | 0.894 (0.045) |
|  |  | $\gamma$ |  | 0.641 (0.024) | 0.642 (0.021) | 1.051 (0.030) | 0.992 (0.026) |  | 0.641 (0.024) | 0.642 (0.021) | 1.051 (0.031) | 0.992 (0.027) |
|  | mSDT | $d^{'}$ |  | 0.496 (0.073) | 0.432 (0.067) | 0.496 (0.074) | 0.436 (0.067) |  | 0.994 (0.069) | 0.874 (0.063) | 0.995 (0.065) | 0.879 (0.060) |
|  |  | $\gamma$ |  | 0.500 (0.031) | 0.502 (0.028) | 0.997 (0.034) | 0.931 (0.030) |  | 0.500 (0.032) | 0.502 (0.028) | 0.997 (0.035) | 0.930 (0.030) |
| 4 | uSDT | $d^{'}$ |  | 0.451 (0.042) | 0.438 (0.040) | 0.477 (0.046) | 0.449 (0.042) |  | 0.893 (0.042) | 0.873 (0.038) | 0.950 (0.043) | 0.896 (0.039) |
|  |  | $\gamma$ |  | 0.869 (0.011) | 0.909 (0.010) | 1.151 (0.018) | 1.145 (0.015) |  | 0.870 (0.012) | 0.909 (0.010) | 1.151 (0.018) | 1.146 (0.015) |
|  | mSDT | $d^{'}$ |  | 0.490 (0.115) | 0.407 (0.091) | 0.492 (0.102) | 0.417 (0.086) |  | 0.991 (0.102) | 0.842 (0.077) | 0.991 (0.080) | 0.857 (0.068) |
|  |  | $\gamma$ |  | 0.504 (0.027) | 0.591 (0.021) | 0.999 (0.026) | 0.991 (0.021) |  | 0.505 (0.027) | 0.592 (0.021) | 0.999 (0.026) | 0.991 (0.021) |
| 6 | uSDT | $d^{'}$ |  | 0.442 (0.043) | 0.441 (0.042) | 0.469 (0.045) | 0.450 (0.042) |  | 0.880 (0.040) | 0.877 (0.039) | 0.934 (0.042) | 0.898 (0.039) |
|  |  | $\gamma$ |  | 1.045 (0.007) | 1.090 (0.006) | 1.241 (0.013) | 1.263 (0.010) |  | 1.046 (0.007) | 1.090 (0.006) | 1.241 (0.013) | 1.263 (0.010) |
|  | mSDT | $d^{'}$ |  | 0.483 (0.173) | 0.391 (0.114) | 0.489 (0.129) | 0.402 (0.102) |  | 0.987 (0.141) | 0.816 (0.092) | 0.989 (0.097) | 0.836 (0.078) |
|  |  | $\gamma$ |  | 0.509 (0.027) | 0.663 (0.018) | 1.002 (0.023) | 1.041 (0.017) |  | 0.509 (0.027) | 0.663 (0.018) | 1.003 (0.023) | 1.041 (0.018) |
| 8 | uSDT | $d^{'}$ |  | 0.441 (0.044) | 0.442 (0.043) | 0.464 (0.046) | 0.451 (0.044) |  | 0.878 (0.040) | 0.882 (0.040) | 0.922 (0.042) | 0.901 (0.039) |
|  |  | $\gamma$ |  | 1.185 (0.004) | 1.225 (0.004) | 1.321 (0.009) | 1.358 (0.008) |  | 1.185 (0.004) | 1.225 (0.004) | 1.321 (0.009) | 1.357 (0.008) |
|  | mSDT | $d^{'}$ |  | 0.473 (0.249) | 0.377 (0.138) | 0.483 (0.162) | 0.389 (0.120) |  | 0.978 (0.199) | 0.798 (0.108) | 0.986 (0.117) | 0.822 (0.087) |
|  |  | $\gamma$ |  | 0.514 (0.029) | 0.723 (0.017) | 1.004 (0.022) | 1.084 (0.015) |  | 0.513 (0.029) | 0.722 (0.016) | 1.005 (0.022) | 0.514 (0.029) |

*Note*. The table displays the means and standard deviations (in parentheses) of discriminability and response criterion estimated from the simulated data. A total of 10,000 simulations were conducted for each combination of model parameters under each lineup size. Each simulation contained 2,000 witness responses, 1,000 for culprit-present lineups and 1,000 for culprit-absent lineups.

Finally, the above simulations assume independence among signals of lineup members. Future research could further explore the model’s ability to estimate parameters when more sophisticated considerations are involved. For example, the uSDT model may be less effective in handling factors such as filler similarity or the number of fillers, whereas the mSDT model provides a more potent tool to address these challenges.

**References Not Included in Main Manuscript**

Banks, W. P. (1970). Signal detection theory and human memory. *Psychological Bulletin*, *74*(2), 81–99. <https://doi.org/10.1037/h0029531>

Friendly, M., Chen, C., Härdle, W. K., & Unwin, A. (2008). A brief history of data visualization. In C. Chen, W. K. Härdle, & A. Unwin (Eds.), *Handbook of data visualization* (pp.15-56). Springer.

Law, A. M., & Kelton, W. D. (2000). *Simulation modeling and analysis*. McGraw-Hill.
